# Changes in sexual behaviors, network attributes, and STI testing among 15–44-year-olds by marital/cohabiting status and partner number: National Survey of Family Growth, 2008-19

**DOI:** 10.1371/journal.pone.0343813

**Published:** 2026-04-02

**Authors:** David A. Katz, Casey E. Copen, Laura T. Haderxhanaj, Matthew Hogben, Deven T. Hamilton

**Affiliations:** 1 Department of Global Health, University of Washington, Seattle, Washington, United States of America; 2 Division of STD Prevention, National Center for HIV/AIDS, Hepatitis, Sexually Transmitted Disease, and Tuberculosis Prevention, Centers for Disease Control and Prevention, Atlanta, Georgia, United States of America; 3 Center for Studies in Demography and Ecology, University of Washington, Seattle, Washington, United States of America; Hospital Femina, BRAZIL

## Abstract

**Background:**

Relationships and recent sexual experience can impact sexual behaviors, networks, and STI testing. How they affect trends in these factors and increasing STI rates is understudied.

**Methods:**

We analyzed data from 28,027 females and 23,479 males ages 15–44 from the National Survey of Family Growth, 2008–2010 through 2017–2019. We used survey-weighted linear or logistic regression to evaluate linear trends in self-reported sexual behaviors with opposite-sex partners; sexual network attributes; and STI testing, stratified by sex and, separately, by marital/cohabiting status and number of past-year vaginal sex partners (1 vs. ≥ 2). Trends p < 0.050 reported below.

**Results:**

From 2008–2019, condom use at last vaginal sex decreased among never-married females (51.9% in 2008–10 to 42.9% in 2017–19), never-married males (61.9%−56.3%), and individuals with ≥2 partners (females = 45.3%−34.9%; males = 54.0%−45.7%). Mean number of vaginal sex acts in past 4 weeks decreased among cohabiting females (9.46–7.40), never-married males (4.11–3.40), and males with 1 partner (7.25–6.62). The proportion of never-married females reporting sex with males with male partner(s) increased from 2.5% to 5.1%; similarly, percentages of never-married males (3.8%−5.5%) and males with ≥2 partners (4.6%−6.3%) reporting sex with males increased. Racial/ethnic homophily with current vaginal sex partners decreased among cohabiting females (86.5%−80.4%) and married and never-married males (married = 88.9%−85.9%; never-married = 81.2%−72.3%). Past-year chlamydia testing increased among females who were married (13.0%−17.1%), previously-married (27.6%−45.8%), or had 1 partner (21.7%−25.4%).

**Conclusions:**

Changes in sexual behaviors, network attributes, and STI testing from 2008–2010 through 2017–2019 varied by marital/cohabiting status and number of past-year opposite-sex partners in complex ways, most commonly among never-married females and males and males with multiple partners. While these changes’ combined potential impact on STI transmission is uncertain, understanding trends in sexual behaviors, networks, and testing by marital/cohabiting status and partner number can contextualize their contributions to the STI epidemic and support sexual health services tailoring and prioritization.

## Introduction

Reported bacterial STI diagnoses, including gonorrhea, chlamydia, and syphilis, increased substantially in the 2010s and early 2020s, reaching their highest rates in the last 30 years with persistent demographic disparities [1]. Recent reports examining sex-, age- and race/ethnicity-stratified trends in sexual behaviors with opposite-sex partners, network attributes, and STI testing among U.S. adolescents and adults from 2008–2019 identified relatively small, complex changes with potentially divergent impacts on both STI transmission and diagnoses [2,3]. Subsequent transmission modeling suggested that key observed changes, including declines in frequency of vaginal sex and condom use and group-specific changes in STI testing, are likely to have accounted for only some of the observed increases in gonorrhea and chlamydia diagnoses nationally from 2012–2019 [4]. However, these studies were limited by lack of attention to how these potential contributors to STI transmission changed within groups defined by relationship status and recent sexual experience, factors that can inform sexual decision-making, network connectivity, health-seeking behaviors, and STI risk [5–11]. And, insofar as small aggregate changes reflect larger changes within specific subgroups, this can have significant implications for both epidemic potential and STI prevention [12]. Using data from the National Survey of Family Growth (NSFG), we conducted an exploratory analysis examining temporal trends in sexual behaviors with opposite-sex partners, network attributes, and STI testing from 2008–2010 through 2017–2019, stratified by marital/cohabiting status and number of opposite-sex partners in the past year, to provide context for how changes in these factors may be contributing to the ongoing STI epidemic and to inform prioritization and tailoring of sexual health services.

## Methods

### Study population

We analyzed data from female and male respondents in the 2008–10, 2011–2013, 2013–2015, 2015–2017, and 2017–2019 NSFG public-use files. NSFG is a nationally-representative survey of the noninstitutionalized, household U.S. population of females and males ages 15–44 through the 2013–2015 data collection, and ages 15–49 in later releases. It uses a multistage area-probability sampling design in which geographic areas (primary sampling units comprise counties or groups of counties and county-equivalents from all U.S. states and the District of Columbia) are first selected, then smaller geographic segments and households within those areas, and finally eligible individuals within sampled households. Sampling rates vary by geography and demographic characteristics, including oversampling Black and Hispanic groups and adolescents aged 15–19, with weights applied to produce nationally representative estimates. For our analysis, the study population was restricted to respondents aged 15–44 for consistency across the 5 survey periods; this excluded 668 (12%) and 728 (12%) of all respondents in the 2015–2017 and 2017–2019 survey periods, respectively. All respondents provided informed consent (ages ≥18 years) or assent after parental permission (ages 15–17). NSFG procedures are approved by the National Center for Health Statistics Research Ethics Review Board and described in detail elsewhere [13–15].

### Measures

#### Key sociodemographics.

Whether respondents were male or female was ascertained through an interviewer-administered screening question. The survey assesses marital/cohabiting status in relation to opposite-sex partners, and we used coding provided by NSFG to define marital/cohabiting status (regardless of partner sex) at the time of the interview as Married; Cohabiting, not married; Divorced, widowed, or separated (i.e., previously married); or Never married.

#### Sexual behaviors.

Measures of penile-vaginal intercourse, concurrency, and racial/ethnic homophily were drawn from face-to-face interviews. Sexual behaviors involving oral and anal sex, having sex with male partners who have sex with men (females) or sex with other men (males), and STI testing were asked via audio computer-assisted self-interview (ACASI).

Sexual behaviors with opposite-sex partners included the following with analysis populations in parentheses: vaginal sex in the past 12 months (respondents reporting ever having vaginal sex); condom use at last vaginal sex and number of vaginal sex acts in the past 4 weeks (vaginal sex in the past 12 months); proportion of condom-protected vaginal sex acts in the past 4 weeks (≥1 vaginal sex act in the past 4 weeks); any oral sex – giving or receiving – or anal sex in the past 12 months (any anal, oral, or vaginal sex in the past 12 months); condom use at last penile-oral sex (any oral sex in the past 12 months and either females ever having given oral sex to a male partner or males ever having received oral sex from a female partner); and condom use at last anal sex (anal sex in the past 12 months). We calculated the proportion of condom-protected vaginal sex acts by dividing the number of condom-protected sex acts by total number of sex acts and assumed that the 33 (0.2%) female respondents who reported more condom-protected than total sex acts in the past 4 weeks had used condoms for all acts.

#### Sexual network attributes.

Sexual network attributes included behaviors and partnership-level characteristics that contribute to linkages among people in sexual networks and were defined for respondents reporting vaginal sex with an opposite-sex partner in the past 12 months. First, we defined having >1 current opposite-sex vaginal sex partner at time of interview (concurrency point prevalence) as either (a) reporting being married to or living with an opposite-sex vaginal sex partner and identified ≥1 additional “current” vaginal sex partner(s) among their most recent 3 partners or (b) reported >1 non-marital, non-cohabiting vaginal sex partner as current. Second, racial/ethnic homophily included up to 3 “current” partnerships in which vaginal sex occurred most recently per respondent and was calculated as the percent of all such partnerships in which both partners are of the same race and ethnicity. However, because NSFG public-use datasets report race and ethnicity as Hispanic, non-Hispanic Black (single race), non-Hispanic White (single race), and non-Hispanic Other or multiple race, we limited analyses of racial and ethnic homophily to the first three groups for interpretability. In addition, partnerships between non-Hispanic Black or White respondents reporting only one race and their multiracial partners were considered non-homophilous, even if one of the partner’s reported races matched that of the respondent, because the public-use dataset categorizes non-Hispanic individuals identifying with multiple races as “Other or multiple race”. Finally, we assessed whether females reported vaginal sex with a male partner in the past 12 months who had ever had sex with other men or males reported oral or anal sex with other men in the past 12 months.

#### STI testing.

Female respondents were asked whether, in the past 12 months, they had tested for chlamydia (all survey periods; “In the last 12 months, that is, since [calculated date], have you been tested for chlamydia?”) and for all other STIs (beginning in 2011–2013; “In the last 12 months, have you been tested for any other sexually transmitted disease like gonorrhea, herpes, or syphilis?”). These measures were combined from 2011–2013 onward to assess testing for any STI. Male respondents were asked only whether they had tested for any STIs (“In the past 12 months, that is, since [calculated date], have you been tested by a doctor or other medical care provider for a sexually transmitted disease like gonorrhea, chlamydia, herpes, or syphilis?”). All testing outcomes were self-reported and analyzed among respondents who reported vaginal sex with an opposite-sex partner in the past 12 months.

### Analyses

We conducted analyses separately for male and female respondents, stratified by marital/cohabiting status and number of opposite-sex vaginal sex partners in the past 12 months (1 vs. ≥ 2). All analyses were weighted to represent the U.S. household population of males and females ages 15–44 at the time of the respective surveys, except for racial/ethnic homophily, which was weighted for their current opposite-sex partnerships using rules for augmented fixed choice design surveys as described previously [2,16].

We evaluated temporal trends using survey-weighted linear or logistic regression. Regression models treated survey year, defined as the mid-point of data collection (June 2008-July 2010; September-September for survey periods 2011–2019), as a linear independent variable (note the break in data collection from July 2010 through September 2011). Resulting β coefficients from linear regression and odds ratios (OR) represent per-year changes from 2009 to 2018 for outcomes available 2008–2017 through 2017–2019 and 2012–2018 for those available 2011–2013 through 2017–2019. In secondary analyses, we adjusted for age and race/ethnicity to account for observed differences in temporal trends across these characteristics in the study’s strata and outcomes [2,3]. Age was categorized as 15–29 vs. 30–44 years for marital/cohabiting status-stratified analyses because almost no 15–19-year-olds were married or formerly married [2,3]. Temporal trends in marital/cohabiting status and partner number were assessed using survey-weighted multinomial and standard logistic regression, respectively.

P-values less than 0.050 were considered statistically significant. For relatively rare behaviors and network attributes (prevalence<3%), the relative size of 95% confidence intervals (95%CI) widths were greater than 130% and should be interpreted with caution. Analyses were conducted using survey analysis procedures in SAS® OnDemand for Academics.

## Results

A total of 28,027 female and 23,479 male respondents ages 15–44 across the five NSFG survey periods from 2008–2010 through 2017–2019 were included in this analysis. Supplemental Tables 1 and 2 present demographic characteristics for females and males, respectively [S1 File].

Proportions, means, regression estimates, and associated 95%CIs from analyses of trends in sexual behaviors, network attributes, and STI testing are presented in Table 1 (females by marital/cohabiting status), Table 2 (females by partner number), Table 3 (males by marital/cohabiting status), and Table 4 (males by partner number).

**Table 1:** Sexual behaviors, network attributes, and STI testing among females, stratified by marital/cohabiting status, in the 2008−10 to 2017−19 survey periods of the National Survey of Family Growth.

Outcome	Marital/cohabiting Status	% or Mean (95% Confidence Interval)					Logistic or linear regression	
		2008−10	2011−13	2013−15	2015−17	2017−19	OR or β (95%CI)	p-value
*Vaginal sex behaviors with male partner(s)*								
Vaginal sex in past 12 months	Married	99.1% (98.5-99.6%)	99.9% (99.7-100.0%)	99.8% (99.6-100.0%)	99.7% (99.4-100.0%)	99.8% (99.5-100.0%)	**1.20** **(1.02-1.41)**	**0.030**
	Cohabiting, not married	99.5% (99.1-99.8%)	99.5% (99.0-100.0%)	99.5% (98.9-100.0%)	99.7% (99.3-100.0%)	98.9% (97.2-100.0%)	0.92 (0.71-1.19)	0.523
	Divorced, widowed, or separated	98.7% (97.3-100.0%)	96.1% (91.6-100.0%)	99.7% (99.2-100.0%)	98.3% (96.5-100.0%)	99.8% (99.5-100.0%)	1.11 (0.98-1.26)	0.098
	Never married	98.0% (97.4-98.6%)	99.7% (99.5-99.9%)	99.0% (98.5-99.5%)	98.7% (98.1-99.4%)	99.3% (98.9-99.7%)	**1.08** **(1.01-1.16)**	**0.032**
Number of vaginal sex acts in past 4 weeks*	Married	7.06 (6.60-7.51)	7.06 (0.59-7.53)	6.99 (6.53-7.46)	7.08 (6.55-7.60)	6.86 (6.35-7.37)	−0.016 (−0.086, 0.054)	0.659
	Cohabiting, not married	9.46 (8.29-10.62)	9.74 (8.74-10.74)	9.14 (8.18-10.10)	8.49 (7.55-9.43)	7.40 (6.70-8.10)	**−0.242** **(−0.384, −0.101)**	**0.0008**
	Divorced, widowed, or separated	7.26 (6.09-8.42)	7.48 (6.46-8.51)	6.03 (5.07-6.99)	7.92 (5.60-10.23)	7.65 (6.24-9.06)	0.037 (−0.164, 0.238)	0.717
	Never married	6.52 (6.04-7.01)	6.44 (5.81-7.07)	6.14 (5.66-6.62)	5.75 (5.05-6.46)	6.52 (5.65-7.39)	−0.033 (−0.136, 0.070)	0.534
Proportion of condom-protected vaginal sex acts in past 4 weeks*	Married	0.157 (0.134-0.180)	0.134 (0.110-0.157)	0.137 (0.114-0.161)	0.151 (0.119-0.184)	0.124 (0.102-0.146)	−0.002 (−0.006, 0.001)	0.191
	Cohabiting, not married	0.212 (0.164-0.260)	0.209 (0.167-0.250)	0.164 (0.122-0.206)	0.146 (0.111-0.181)	0.146 (0.103-0.190)	**−0.009** **(−0.015, −0.002)**	**0.008**
	Divorced, widowed, or separated	0.223 (0.130-0.316)	0.300 (0.217-0.375)	0.217 (0.135-0.300)	0.188 (0.106-0.270)	0.282 (0.197-0.366)	0.001 (−0.012, 0.014)	0.873
	Never married	0.531 (0.489-0.573)	0.431 (0.388-0.475)	0.426 (0.385-0.467)	0.386 (0.336-0.435)	0.378 (0.321-0.435)	**−0.016** **(−0.024, −0.009)**	**<0.0001**
Condom use at last vaginal sex	Married	15.5% (12.9-18.0%)	13.1% (11.1-15.2%)	15.2% (12.9-17.5%)	15.6% (12.0-19.2%)	14.3% (11.6-17.0%)	1.00 (0.97-1.03)	0.954
	Cohabiting, not married	19.5% (15.8-23.3%)	21.0% (17.6-24.5%)	18.1% (13.9-22.3%)	15.5% (11.5-19.6%)	18.0% (14.2-21.9%)	0.98 (0.94-1.01)	0176
	Divorced, widowed, or separated	26.9% (18.9-34.9%)	35.2% (28.1-42.3%)	24.0% (18.1-29.8%)	29.5% (22.5-36.5%)	28.7% (22.7-34.6%)	1.00 (0.95-1.05)	0.965
	Never married	51.9% (47.5-56.3%)	49.1% (46.0-52.2%)	46.9% (42.8-50.9%)	41.7% (38.2-45.2%)	42.9% (38.1-47.7%)	**0.96** **(0.93-0.98)**	**0.0005**
								
*Oral and anal sex behaviors with male partner(s)*†‡								
Had oral sex in past 12 months	Married	NA	83.8% (81.6-86.0%)	83.9% (81.0-86.7%)	81.7% (78.0-85.4%)	86.8% (84.6-89.0%)	1.03 (0.99-1.07)	0.231
	Cohabiting, not married	NA	88.3% (85.5-91.1%)	87.6% (84.4-90.9%)	87.6% (83.7-91.6%)	91.7% (88.4-95.1%)	1.05 (0.98-1.13)	0.159
	Divorced, widowed, or separated	NA	85.2% (79.9-90.6%)	87.7% (82.4-92.9%)	91.4% (88.0-94.8%)	92.4% (89.5-95.2%)	**1.14** **(1.04-1.26)**	**0.008**
	Never married	NA	85.6% (82.2-88.9%)	87.4% (85.2-89.6%)	89.3% (86.9-91.8%)	90.7% (88.3-93.1%)	**1.09** **(1.02-1.16)**	**0.008**
Condom use at last oral sex	Married	NA	3.8% (2.2-5.5%)	1.7% (1.0-2.3%)	2.1% (1.4-2.9%)	2.0% (1.2-2.9%)	0.90 (0.80-1.01)	0.071
	Cohabiting, not married	NA	4.9% (2.8-7.1%)	4.1% (2.3-6.0%)	3.7% (2.0-5.5%)	3.4% (1.1-5.8%)	0.94 (0.82-1.07)	0.338
	Divorced, widowed, or separated	NA	11.1% (4.8-17.4%)	8.0% (3.4-12.7%)	5.3% (0.8-9.8%)	8.0% (3.7-12.4%)	0.92 (0.78-1.08)	0.289
	Never married	NA	10.0% (7.4-12.6%)	8.1% (6.1-10.1%)	6.6% (4.7-8.6%)	7.1% (4.5-9.7%)	0.93 (0.86-1.01)	0.094
Had anal sex in past 12 months	Married	NA	19.0% (16.0-21.9%)	18.4% (15.9-20.9%)	19.1% (16.0-22.1%)	21.9% (18.7-25.0%)	1.03 (0.99-1.08)	0.175
	Cohabiting, not married	NA	27.1% (21.5-32.8%)	28.9% (25.0-32.8%)	27.9% (21.3-34.5%)	29.0% (22.9-35.1%)	1.01 (0.95-1.08)	0.728
	Divorced, widowed, or separated	NA	24.8% (18.7-30.9%)	35.4% (28.6-42.3%)	27.8% (18.6-37.3%)	25.7% (20.7-30.7%)	0.99 (0.93-1.06)	0.858
	Never married	NA	19.2% (16.0-22.5%)	20.4% (18.0-22.8%)	18.7% (15.6-21.8%)	21.5% (17.9-25.0%)	1.02 (0.97-1.07)	0.501
Condom use at last anal sex in past 12 months	Married	NA	9.2% (4.9-13.4%)	13.5% (8.1-19.0%)	11.3% (6.6-16.0%)	15.5% (8.5-22.4%)	1.08 (0.96-1.22)	0.190
	Cohabiting, not married	NA	12.5% (7.1-17.8%)	13.6% (8.5-18.7%)	11.2% (2.5-19.9%)	14.8% (6.1-23.5%)	1.02 (0.89-1.18)	0.767
	Divorced, widowed, or separated	NA	17.4% (8.9-25.9%)	17.5% (8.1-26.9%)	25.6% (9.4-41.8%)	25.7% (12.6-38.9%)	1.11 (0.96-1.29)	0.170
	Never married	NA	27.7% (18.6-36.7%)	26.9% (20.2-33.6%)	25.0% (16.7-33.3%)	23.0% (15.9-30.2%)	0.96 (0.87-1.05)	0.377
								
*Sexual network attributes*								
Vaginal, oral, or anal sex with a man who has sex with men in past 12 months	Married	1.1% (0.59-1.67)	1.6% (0.7-2.5%)	0.7% (0.4-1.1%)	0.6% (0.2-1.0%)	1.5% (0.8-2.2%)	0.98 (0.91-1.07)	0.702
	Cohabiting, not married	2.0% (0.6-3.5%)	1.7% (0.6-2.9%)	1.8% (0.5-3.1%)	2.6% (0.0-5.2%)	2.1% (0.7-3.6%)	1.02 (0.92-1.15)	0.674
	Divorced, widowed, or separated	4.9% (0.8-9.1%)	2.6% (0.8-4.4%)	3.0% (0.9-5.0%)	4.5% (2.3-6.6%)	4.5% (1.1-8.0%)	1.00 (0.87-1.15)	0.989
	Never married	2.5% (1.7-3.4%)	2.3% (1.3-3.3%)	3.9% (2.6-5.3%)	3.4% (1.8-5.1%)	5.1% (2.9-7.3%)	**1.09** **(1.02-1.17)**	**0.008**
Racial/ethnic homophily (among up to 3 current male vaginal sex partners)^	Married	89.4% (87.5-91.4%)	87.0% (84.4-89.7%)	87.4% (85.0-89.9%)	87.6% (85.0-90.2%)	87.9% (85.1-90.7%)	0.99 (0.96-1.02)	0.407
	Cohabiting, not married	86.5% (83.7-89.3%)	84.2% (79.9-88.5%)	83.7% (79.9-87.6%)	80.4% (73.1-87.7%)	80.4% (75.5-85.4%)	**0.95** **(0.91-0.99)**	**0.023**
							
	Divorced, widowed, or separated	87.1% (81.6-92.5%)	73.7% (65.9-81.5%)	76.4% (67.1-85.7%)	78.4% (68.9-87.9%)	83.7% (78.0-89.4%)	0.97 (0.92-1.03)	0.361
	Never married	80.7% (76.8-84.7%)	78.6% (74.6-82.7%)	77.4% (73.4-81.3%)	80.7% (75.9-85.4%)	77.0% (72.1-81.8%)	0.99 (0.95-1.02)	0.424
Concurrency (≥2 current male vaginal sex partners at time of survey)	Married	0.4% (0.1-0.8%)	0.5% (0.0-1.0%)	0.2% (0.0-0.5%)	1.1% (0.1-2.1%)	1.0% (0.4-1.5%)	1.13 (1.0-1.28)	0.050
	Cohabiting, not married	0.6% (0.2-1.1%)	0.8% (0.2-1.4%)	0.4% (0.0-0.8%)	1.0% (0.0-2.1%)	0.1% (0.0-0.3%)	0.94 (0.83-1.07)	0.365
	Divorced, widowed, or separated	1.5% (0.0-3.0%)	3.6% (1.3-5.9%)	0.9% (0.2-1.6%)	1.2% (0.3-2.0%)	3.5% (0.3-6.6%)	1.03 (0.89-1.19)	0.728
	Never married	2.1% (1.4-2.8%)	1.9% (1.2-2.6%)	1.8% (1.2-2.5%)	1.9% (1.2-2.7%)	1.9% (0.7-3.1%)	0.99 (0.92-1.06)	0.779
								
*STI Testing*								
Chlamydia testing in past 12 months	Married	13.0% (11.3-14.7%)	14.9% (12.7-17.1%)	15.1% (12.7-17.5%)	17.6% (14.8-20.4%)	17.1% (14.6-19.7%)	**1.04** **(1.02-1.07)**	**0.002**
	Cohabiting, not married	31.8% (26.6-36.9%)	32.8% (26.9-38.6%)	37.1% (33.6-40.6%)	37.7% (32.1-43.3%)	30.7% (25.8-35.5%)	1.01 (0.97-1.04)	0.757
	Divorced, widowed, or separated	27.6% (21.2-34.0%)	37.5% (30.8-44.1%)	40.0% (31.6-48.3%)	38.8% (30.9-46.7%)	45.8% (39.2-52.5%)	**1.08** **(1.04-1.13)**	**0.0005**
	Never married	46.2% (42.3-50.0%)	42.7% (37.5-47.8%)	49.6% (46.3-52.8%)	48.5% (44.5-52.4%)	45.1% (40.1-50.0%)	1.01 (0.98-1.03)	0.667
STI testing in past 12 months†	Married	NA	18.8% (16.5-21.2%)	19.9% (17.3-22.5%)	23.3% (20.2-26.4%)	21.1% (17.9-24.2%)	1.03 (0.99-1.07)	0.105
	Cohabiting, not married	NA	36.3% (30.5-42.1%)	43.1% (39.0-47.2%)	43.6% (38.0-49.2%)	38.6% (33.6-43.6%)	1.02 (0.96-1.07)	0.574
	Divorced, widowed, or separated	NA	42.6% (35.6-49.6%)	45.6% (38.4-52.8%)	46.4% (38.2-54.6%)	56.0% (49.6-62.4%)	**1.08** **(1.02-1.15)**	**0.014**
	Never married	NA	49.7% (45.0-54.3%)	55.7% (52.3-59.2%)	54.0% (50.5-57.6%)	50.9% (46.1-55.7%)	1.00 (0.96-1.05)	0.896

Bold indicates significance at <0.05 level.

*Estimates derived from linear regression (β). All others from logistic regression (odds ratios; OR). All models accounted for survey weights.

^Measured among Hispanic, Non-Hispanic Black, and Non-Hispanic White individuals only.

†Analysis includes 2011−13–2017−19 survey periods only. In the 2008−10 survey period, female respondents were not asked about STI testing other than chlamydia, and the questions about numbers of opposite-sex partners in the past 12 months for any type of sex and for specific acts were only asked if respondents reported more than 1 lifetime vaginal sex partner in the interviewer-administered portion of the survey, which led to ~20% missingness and significant bias for estimates of total, vaginal, oral, and anal sex numbers in the past 12 months.

**Table 2:** Sexual behaviors, network attributes, and STI testing among females, stratified by number of male vaginal sex partners in the past 12 months, in the 2008−10 to 2017−19 survey periods of the National Survey of Family Growth.

Outcome	Male partners in past 12 months	% or Mean (95% Confidence Interval)					Logistic or linear regression	
		2008−10	2011−13	2013−15	2015−17	2017−19	OR or β (95%CI)	p-value
*Vaginal sex behaviors with male partner(s)*								
Number of vaginal sex acts in past 4 weeks*	1 partner	7.25 (6.85-7.65)	7.37 (6.96-7.77)	7.03 (6.69-7.36)	6.95 (6.55-7.35)	6.62 (6.25-7.00)	**−0.073** **(−0.129, −0.016)**	**0.012**
	≥2 partners	8.04 (7.00-9.08)	8.18 (6.99-9.37)	7.98 (7.01-8.95)	7.58 (6.27-8.89)	8.69 (7.27-10.11)	0.026 (−0.152, 0.204)	0.773
Proportion of condom-protected vaginal sex acts in past 4 weeks*	1 partner	0.220 (0.197-0.243)	0.198 (0.178-0.217)	0.188 (0.167-0.209)	0.184 (0.153-0.215)	0.181 (0.159-0.203)	**−0.004** **(−0.008, −0.001)**	**0.013**
	≥2 partners	0.452 (0.394-0.509)	0.361 (0.313-0.408)	0.348 (0.295-0.401)	0.342 (0.277-0.406)	0.298 (0.234-0.362)	**−0.015** **(−0.024, −0.007)**	**0.0006**
Condom use at last vaginal sex	1 partner	23.7% (21.3-26.1%)	23.3% (21.3-25.2%)	22.2% (19.9-24.4%)	22.0% (19.2-24.9%)	22.6% (20.1-25.2%)	0.99 (0.97-1.01)	0.386
	≥2 partners	45.3% (40.1-50.5%)	39.3% (34.8-43.7%)	38.4% (33.8-43.1%)	34.4% (28.4-40.5%)	34.9% (29.1-40.7%)	**0.95** **(0.92-0.98)**	**0.003**
								
*Oral and anal sex behaviors with male partner(s)*†‡								
Had oral sex in past 12 months	1 partner	NA	84.1% (82.2-86.0%)	84.7% (82.8-86.6%)	83.8% (81.1-86.4%)	87.5% (85.8-89.2%)	**1.04** **(1.00-1.07)**	**0.034**
	≥2 partners	NA	91.1% (89.4-92.9%)	90.3% (87.3-93.3%)	93.0% (90.1-95.9%)	97.6% (96.6-98.7%)	**1.19** **(1.11-1.27)**	**<0.0001**
Condom use at last oral sex	1 partner	NA	6.1% (4.6-7.6%)	3.7% (3.0-4.5%)	3.3% (2.4-4.2%)	3.9% (2.9-4.9%)	**0.92** **(0.86-0.98)**	**0.012**
	≥2 partners	NA	7.4% (5.2-9.7%)	7.8% (5.1%−10.5%)	6.4% (3.9-8.8%)	5.8% (2.6-9.0%)	0.95 (0.86-1.05)	0.324
Had anal sex in past 12 months	1 partner	NA	20.2% (18.0-22.4%)	20.4% (18.6-22.1%)	20.1% (17.8-22.4%)	21.9% (19.6-24.3%)	1.02 (0.98-1.05)	0.339
	≥2 partners	NA	26.8% (22.1-31.4%)	31.7% (27.2-36.2%)	27.6% (22.5-32.8%)	31.4% (25.6-37.2%)	1.02 (0.97-1.08)	0.406
Condom use at last anal sex in past 12 months	1 partner	NA	13.8% (9.8-17.9%)	14.8% (11.5-18.1%)	14.9% (10.1-19.8%)	17.7% (13.4-22.0%)	1.05 (0.98-1.13)	0.205
	≥2 partners	NA	21.6% (14.4-28.8%)	23.4% (16.2-30.5%)	20.3% (11.9-28.6%)	19.2% (13.4-25.1%)	0.97 (0.89-1.06)	0.483
								
*Sexual network attributes*								
Vaginal, oral, or anal sex with a man who has sex with men in past 12 months	1 partner	1.5% (1.0-2.1%)	1.6% (1.0-2.2%)	1.2% (0.8-1.6%)	1.7% (0.9-2.5%)	1.9% (1.2-2.6%)	1.02 (0.96-1.09)	0.449
	≥2 partners	4.2% (2.3-6.2%)	3.4% (1.7-5.1%)	5.6% (3.2-8.0%)	3.8% (2.3-5.4%)	8.0% (4.0-12.0%)	1.08 (0.99-1.17)	0.100
Racial/ethnic homophily (among up to 3 current male vaginal sex partners)^	1 partner	87.5% (85.6-89.4%)	85.5% (83.2-87.7%)	85.7% (83.9-87.5%)	84.7% (82.0-87.5%)	84.6% (82.1-87.2%)	0.98 (0.95-1.00)	0.062
	≥2 partners	85.0% (82.5-87.4%)	74.3% (68.9-79.7%)	73.1% (66.2-80.0%)	80.9% (75.3-86.5%)	76.0% (70.2-81.8%)	0.97 (0.93-1.01)	0.086
Concurrency (≥2 current male vaginal sex partners at time of survey)	1 partner	NA	NA	NA	NA	NA	NA	NA
	≥2 partners	6.1% (4.4-7.8%)	5.9% (4.2-7.6%)	4.3% (2.9-5.7%)	6.7% (4.0-9.5%)	6.6% (3.7-9.5%)	1.02 (0.96-1.08)	0.628
								
*STI Testing*								
Chlamydia testing in past 12 months	1 partner	21.7% (19.7-23.8%)	23.7% (21.4-25.9%)	25.7% (23.6-27.8%)	27.5% (25.3-29.6%)	25.4% (23.1-27.7%)	**1.03** **(1.01-1.05)**	**0.001**
	≥2 partners	50.5% (46.8-54.2%)	46.3% (40.2-52.5%)	53.3% (48.9-58.7%)	49.8% (45.1-54.4%)	53.2% (47.1-59.3%)	1.01 (0.99-1.04)	0.320
STI testing in past 12 months†	1 partner	NA	28.2% (25.8-30.6%)	31.1% (28.7-33.6%)	32.5% (30.3-37.7%)	30.9% (28.1-33.7%)	1.02 (1.00-1.05)	0.109
	≥2 partners	NA	52.4% (46.5-58.3%)	58.8% (54.1-63.5%)	58.5% (53.8-63.3%)	59.2% (53.0-65.4%)	1.04 (0.99-1.10)	0.133

Bold indicates significance at <0.05 level.

*Estimates derived from linear regression (β). All others from logistic regression (odds ratios; OR). All models accounted for survey weights.

^Measured among Hispanic, Non-Hispanic Black, and Non-Hispanic White individuals only.

†Analysis includes 2011−13–2017−19 survey periods only. In the 2008−10 survey period, female respondents were not asked about STI testing other than chlamydia, and the questions about numbers of opposite-sex partners in the past 12 months for any type of sex and for specific acts were only asked if respondents reported more than 1 lifetime vaginal sex partner in the interviewer-administered portion of the survey, which led to ~20% missingness and significant bias for estimates of total, vaginal, oral, and anal sex numbers in the past 12 months.

**Table 3:** Sexual behaviors, network attributes, and STI testing among males, stratified by marital/cohabiting status, in the 2008−10 to 2017−19 survey periods of the National Survey of Family Growth.

Outcome	Marital/cohabiting Status	% or Mean (95% Confidence Interval)					Logistic or linear regression	
		2008−10	2011−13	2013−15	2015−17	2017−19	OR or β (95%CI)	p-value
*Vaginal sex behaviors with female partner(s)*								
Vaginal sex in past 12 months	Married	99.8% (99.6-100.0%)	99.3% (98.3-100.0%)	99.4% (98.9-99.8%)	99.9% (99.7-100.0%)	99.2% (98.4-100.0%)	0.94 (0.79-1.11)	0.453
	Cohabiting, not married	98.6% (97.4-99.8%)	99.1% (98.3-99.9%)	99.9% (99.7-100.0%)	99.9% (99.8-100.0%)	98.9% (97.9-99.9%)	1.11 (0.91-1.36)	0.301
	Divorced, widowed, or separated	99.4% (98.8-100.0%)	99.3% (98.7-99.9%)	99.3% (98.5-100.0%)	99.7% (99.2-100.0%)	99.8% (99.3-100.0%)	1.09 (0.92-1.28)	0.324
	Never married	97.4% (96.1-98.6%)	97.9% (96.7-99.0%)	98.7% (98.1-99.3%)	98.9% (98.4-99.4%)	99.0% (98.4-99.5%)	**1.13** **(1.05-1.22)**	**0.002**
Number of vaginal sex acts in past 4 weeks*	Married	5.60 (5.19-6.01)	6.28 (5.85-6.72)	6.22 (5.79-6.64)	6.34 (5.88-6.79)	5.43 (4.97-5.89)	−0.002 (−0.066, 0.062)	0.953
	Cohabiting, not married	9.41 (7.64-11.19)	9.17 (8.16-10.18)	9.16 (8.16-10.16)	7.95 (7.06-8.83)	7.92 (6.75-9.09)	−0.186 (−0.396, 0.023)	0.081
	Divorced, widowed, or separated	4.68 (3.29-6.07)	5.63 (4.18-7.07)	6.28 (4.57-7.99)	4.98 (3.33-6.63)	5.43 (3.95-6.90)	0.062 (−0.147, 0.271)	0.561
	Never married	4.11 (3.66-4.55)	4.21 (3.56-4.85)	4.00 (3.57-4.42)	3.91 (3.24-4.57)	3.40 (3.02-3.79)	**−0.075** **(−0.142, −0.008)**	**0.029**
Proportion of condom-protected vaginal sex acts in past 4 weeks*	Married	0.156 (0.125-0.187)	0.146 (0.117-0.174)	0.142 (0.112-0.171)	0.136 (0.106-0.165)	0.129 (0.103-0.156)	−0.003 (−0.007, 0.001)	0.171
	Cohabiting, not married	0.224 (0.173-0.274)	0.239 (0.187-0.291)	0.223 (0.174-0.272)	0.199 (0.148-0.249)	0.197 (0.138-0.256)	−0.004 (−0.012, 0.004)	0.306
	Divorced, widowed, or separated	0.400 (0.277-0.516)	0.360 (0.292-0.429)	0.347 (0.270-0.425)	0.359 (0.2.51-0.466)	0.445 (0.326-0.564)	0.002 (−0.014, 0.019)	0.780
	Never married	0.588 (0.550-0.625)	0.605 (0.565-0.645)	0.587 (0.547-0.627)	0.506 (0.462-0.551)	0.519 (0.469-0.570)	**−0.010** **(−0.017, −0.004)**	**0.001**
Condom use at last vaginal sex	Married	17.4% (14.1-20.6%)	20.3% (16.7-23.9%)	16.5% (13.7-19.2%)	17.5% (13.7-21.3%)	15.0% (12.1-17.8%)	0.98 (0.95-1.01)	0.190
	Cohabiting, not married	25.9% (21.7-30.0%)	25.7% (20.2-31.3%)	24.3% (19.6-29.0%)	23.5% (17.8-29.2%)	24.4% (18.4-30.3%)	0.99 (0.95-1.03)	0.528
	Divorced, widowed, or separated	45.2% (36.2-54.2%)	34.2% (28.2-40.1%)	40.9% (34.3-47.6%)	40.6% (31.0-50.2%)	45.2% (35.8-54.6%)	1.01 (0.95-1.06)	0.850
	Never married	61.9% (58.6-65.2%)	65.0% (61.6-68.4%)	64.0% (60.7-67.4%)	56.7% (52.4-61.0%)	56.3% (52.3-60.3%)	**0.97** **(0.94-0.99)**	**0.002**
								
*Oral and anal sex behaviors with female partner(s)*†								
Had oral sex in past 12 months	Married	NA	85.0% (82.2-87.7%)	83.1% (79.6-86.6%)	86.8% (84.2-89.3%)	83.7% (81.1-86.2%)	1.00 (0.95-1.05)	0.989
	Cohabiting, not married	NA	87.5% (84.2-90.8%)	91.0% (88.0-93.9%)	90.4% (86.5-94.3%)	93.2% (90.6-95.8%)	**1.10** **(1.02-1.20)**	**0.019**
	Divorced, widowed, or separated	NA	91.8% (87.9-95.7%)	90.2% (84.4-96.1%)	93.9% (88.8-99.0%)	96.9% (93.8-100.0%)	**1.15** **(1.00-1.33)**	**0.0499**
	Never married	NA	88.3% (85.1-91.5%)	90.8% (89.3-92.4%)	90.3% (88.2-92.4%)	90.7% (88.5-92.9%)	1.04 (0.97-1.11)	0.269
Condom use at last oral sex	Married	NA	3.4% (1.4-5.3%)	3.1% (1.3-4.8%)	2.3% (1.3-3.3%)	1.2% (0.6-1.8%)	**0.86** **(0.77-0.97)**	**0.012**
	Cohabiting, not married	NA	4.1% (1.3-6.9%)	5.5% (2.8-8.1%)	3.1% (1.1-5.2%)	4.6% (2.3-7.0%)	0.99 (0.86-1.14)	0.891
	Divorced, widowed, or separated	NA	7.6% (4.1-11.2%)	6.4% (2.4-10.4%)	15.7% (5.8-25.5%)	4.7% (1.9-7.5%)	1.00 (0.89-1.13)	0.944
	Never married	NA	9.4% (7.5-11.3%)	9.5% (7.1-11.9%)	8.1% (6.3-10.0%)	7.8% (5.7-9.9%)	0.96 (0.91-1.02)	0.176
Had anal sex in past 12 months	Married	NA	20.4% (16.3-24.5%)	19.3% (16.5-22.1%)	22.5% (18.6-26.4%)	22.4% (19.2-25.6%)	1.03 (0.98-1.08)	0.261
	Cohabiting, not married	NA	33.5% (27.6-39.4%)	31.3% (25.8-36.7%)	28.0% (22.6-33.3%)	36.6% (30.1-43.1%)	1.01 (0.95-1.08)	0.667
	Divorced, widowed, or separated	NA	37.1% (28.8-46.3%)	38.5% (31.3-45.8%)	34.6% (26.4-42.8%)	35.0% (27.2-42.8%)	0.98 (0.90-1.07)	0.628
	Never married	NA	23.2% (19.7-26.9%)	22.4% (19.3-25.5%)	20.9% (17.4-24.3%)	17.7% (14.9-20.5%)	**0.95** **(0.91-0.99)**	**0.013**
Condom use at last anal sex in past 12 months	Married	NA	19.2% (12.3-26.0%)	21.1% (13.5-28.7%)	19.4% (12.4-26.3%)	19.6% (13.5-25.7%)	1.00 (0.91-1.10)	0.968
	Cohabiting, not married	NA	24.1% (15.6-32.6%)	17.8% (10.1-25.6%)	34.8% (22.0-47.6%)	22.3% (13.2-31.3%)	1.02 (0.92-1.14)	0.688
	Divorced, widowed, or separated	NA	23.4% (10.6-36.1%)	23.1% (13.0-33.3%)	30.0% (16.8-43.2%)	29.9% (16.3-43.5%)	1.07 (0.92-1.25)	0.389
	Never married	NA	43.4% (36.6-50.3%)	41.7% (33.6-49.7%)	35.4% (27.8-43.0%)	41.7% (32.8-50.6%)	0.97 (0.91-1.05)	0.463
								
*Sexual network attributes*								
Ever had oral or anal sex with another man	Married	3.3% (2.3-4.3%)	3.9% (2.6-5.2%)	3.2% (1.9-4.5%)	3.9% (2.3-5.4%)	4.7% (3.5-5.9%)	1.04 (0.99-1.08)	0.152
	Cohabiting, not married	4.1% (2.0-6.2%)	5.5% (3.0-8.1%)	3.1% (3.0-8.1%)	1.8% (0.5-3.1%)	5.1% (2.4-7.8%)	0.98 (0.90-1.07)	0.635
	Divorced, widowed, or separated	2.0% (0.4-3.7%)	4.2% (1.8-6.6%)	5.0% (1.3-8.6%)	4.0% (0.0-8.0%)	6.3% (2.0-10.6%)	1.11 (1.00-1.23)	0.057
	Never married	3.8% (2.6-5.0%)	4.4% (2.7-6.2%)	4.2% (3.0-5.4%)	6.1% (3.7-8.5%)	5.5% (3.9-7.1%)	**1.05** **(1.00-1.11)**	**0.041**
Racial/ethnic homophily (among up to 3 current female vaginal sex partners)^	Married	88.9% (86.2-91.6%)	86.7% (83.2-90.0%)	85.6% (82.6-88.6%)	78.8% (74.5-83.1%)	85.9% (82.4-89.4%)	**0.95** **(0.91-0.99)**	**0.006**
	Cohabiting, not married	84.4% (81.1-87.6%)	81.6% (77.1-86.2%)	83.6% (78.8-88.4%)	83.0% (77.3-88.7%)	77.4% (71.7-83.1%)	0.96 (0.92-1.01)	0.093
	Divorced, widowed, or separated	87.2% (80.7-93.8%)	82.5% (75.1-89.9%)	84.3% (78.1-90.5%)	78.4% (69.2-87.6%)	76.4% (67.3-85.5%)	0.93 (0.89-1.00)	0.050
	Never married	81.2% (77.4-85.0%)	77.3% (72.7-81.9%)	77.1% (71.2-83.0%)	78.1% (74.3-81.9%)	72.3% (67.8-76.7%)	**0.96** **(0.92-0.99)**	**0.011**
Concurrency (≥2 current female vaginal sex partners at time of survey)	Married	0.9% (0.3-1.4%)	0.4% (0.2-0.6%)	0.5% (0.0-1.0%)	0.7% (0.1-1.2%)	0.1% (0.0-0.3%)	0.90 0.80-1.02)	0.097
	Cohabiting, not married	3.6% (0.4-6.8%)	1.6% (0.0-3.7%)	5.1% (2.0-8.3%)	3.0% (0.2-5.7%)	2.4% (0.5-4.3%)	0.98 (0.87-1.11)	0.780
	Divorced, widowed, or separated	12.4% (7.0-17.8%)	14.8% (9.9-19.7%)	6.8% (3.4-10.2%)	6.7% (1.9-11.6%)	7.3% (2.5-12.2%)	**0.91** **(0.84-0.99)**	**0.029**
	Never married	3.4% (2.1-4.7%)	3.2% (2.3-4.1%)	3.5% (2.4-4.6%)	2.8% (1.7-3.9%)	2.1% (1.1-3.1%)	0.96 (0.91-1.01)	0.106
								
*STI Testing*								
STI testing in past 12 months	Married	8.4% (6.8-10.0%)	8.6% (5.8-11.4%)	8.7% (6.5-10.8%)	11.8% (8.7-15.0%)	9.0% (6.8-11.1%)	1.02 (0.99-1.06)	0.184
	Cohabiting, not married	21.4% (15.1-27.7%)	20.6% (15.4-25.7%)	14.7% (10.8-18.7%)	13.6% (8.8-18.4%)	16.2% (11.5-20.8%)	**0.95** **(0.90-1.00)**	**0.043**
	Divorced, widowed, or separated	26.5% (19.3-33.8%)	22.4% (15.5-29.4%)	22.9% (17.1-28.7%)	26.7% (18.9-34.6%)	21.4% (15.4-27.5%)	0.98 (0.93-1.04)	0.535
	Never married	24.6% (21.4-27.8%)	26.2% (23.5-29.0%)	26.1% (23.5-28.7%)	24.5% (21.0-28.0%)	21.9% (17.8-26.0%)	0.98 (0.96-1.01)	0.258

Bold indicates significance at <0.05 level.

*Estimates derived from linear regression (β). All others from logistic regression (odds ratios; OR). All models accounted for survey weights.

^Measured among Hispanic, Non-Hispanic Black, and Non-Hispanic White individuals only.

†Analysis includes 2011−13–2017−19 survey periods only. In the 2008−10 survey period, the questions about numbers of opposite-sex partners in the past 12 months for any type of sex and for specific acts were only asked if respondents reported more than 1 lifetime vaginal sex partner in the interviewer-administered portion of the survey, which led to ~20% missingness and significant bias for estimates of total, vaginal, oral, and anal sex numbers in the past 12 months.

**Table 4:** Sexual behaviors, network attributes, and STI testing among males, stratified by number of female vaginal sex partners in the past 12 months, in the 2008−10 to 2017−19 survey periods of the National Survey of Family Growth.

Outcome	Marital/cohabiting Status	% or Mean (95% Confidence Interval)					Logistic or linear regression	
		2008−10	2011−13	2013−15	2015−17	2017−19	OR or β (95%CI)	p-value
*Vaginal sex behaviors with female partner(s)*								
Number of vaginal sex acts in past 4 weeks*	1 partner	5.52 (5.18-5.85)	6.04 (5.69-6.40)	6.08 (5.72-6.44)	5.80 (5.44-6.15)	5.21 (4.78-5.63)	−0.032 (−0.086, 0.022)	0.245
	≥2 partners	6.01 (4.78-7.24)	5.89 (5.02-6.77)	5.64 (4.73-6.55)	5.29 (4.24-6.34)	4.96 (4.27-5.66)	−0.118 (−0.268, 0.032)	0.123
Proportion of condom-protected vaginal sex acts in past 4 weeks*	1 partner	0.229 (0.203-0.254)	0.228 (0.200-0.256)	0.214 (0.188-0.239)	0.202 (0.174-0.229)	0.222 (0.191-0.253)	−0.002 (−0.006, 0.002)	0.369
	≥2 partners	0.516 (0.477-0.554)	0.541 (0.492-0.590)	0.516 (0.465-0.566)	0.456 (0.397-0.515)	0.441 (0.391-0.491)	**−0.010** **(−0.016, −0.003)**	**0.004**
Condom use at last vaginal sex	1 partner	29.3% (26.9-31.6%)	31.8% (29.3-34.3%)	28.4% (25.9-30.9%)	29.2% (25.5-32.9%)	29.4% (26.3-32.4%)	1.00 (0.98-1.02)	0.645
	≥2 partners	54.0% (50.0-58.0%)	55.7% (51.0-60.3%	55.1% (51.0-59.2%)	47.0% (40.8-53.1%)	45.7% (40.6-50.7%)	**0.96** **(0.93-0.99)**	**0.002**
								
*Oral and anal sex behaviors with female partner(s)*†‡								
Had oral sex in past 12 months	1 partner	NA	85.0% (82.5-87.5%)	84.3% (81.9-86.7%)	87.1% (85.3-88.9%)	86.7% (85.0-88.3%)	.1.03 (0.99-1.07)	0.107
	≥2 partners	NA	93.7% (91.5-96.0%)	96.9% (96.7-98.1%)	95.4% (92.9-97.8%)	96.4% (94.7-98.0%)	1.08 (0.97-1.20)	0.160
Condom use at last oral sex	1 partner	NA	4.8% (3.4-6.2%)	4.9% (3.8-6.0%)	4.6% (3.1-6.1%)	3.5% (2.6-4.3%)	0.95 (0.89-1.01)	0.112
	≥2 partners	NA	9.5% (6.4-12.6%)	8.0% (5.5-10.5%)	6.5% (3.8-9.1%)	6.7% (3.9-9.5%)	0.93 (0.85-1.02)	0.131
Had anal sex in past 12 months	1 partner	NA	21.3% (18.2-24.4%)	21.1% (18.9-23.3%)	22.7% (19.8-25.5%)	22.5% (19.5-25.5%)	1.02 (0.98-1.06)	0.464
	≥2 partners	NA	37.0% (32.3-41.8%)	32.4% (27.1-37.7%)	26.5% (22.7-30.2%)	29.4% (24.5-34.3%)	**0.93** **(0.89-0.98)**	**0.008**
Condom use at last anal sex in past 12 months	1 partner	NA	22.5% (17.6-27.3%)	22.9% (17.8-28.1%)	24.2% (18.4-29.9%)	25.9% (21.3-30.5%)	1.03 (0.97-1.10)	0.288
	≥2 partners	NA	39.4% (32.4-46.5%)	38.0% (29.9-46.1%)	39.9% (30.1-49.7%)	30.7% (22.7-38.8%)	0.95 (0.88-1.03)	0.186
								
*Sexual network attributes*								
Ever had oral or anal sex with another man	1 partner	3.2% (2.4-4.1%)	4.5% (3.2-5.7%)	3.0% (2.3-3.8%)	3.4% (2.3-4.5%)	4.9% (3.9-5.9%)	1.03 (0.99-1.07)	0.159
	≥2 partners	4.6% (3.2-6.0%)	4.2% (2.3-6.2%)	5.1% (3.2-7.0%)	8.1% (4.6-11.5%)	6.3% (4.2-8.4%)	**1.06** **(1.01-1.12)**	**0.026**
Racial/ethnic homophily (among up to 3 current female vaginal sex partners)^	1 partner	87.3% (85.1-89.5%)	84.9% (82.4-87.4%)	84.1% (81.5-86.7%)	80.0% (76.7-83.4%)	81.1% (78.4-83.8%)	**0.94** **(0.92-0.97)**	**<0.001**
	≥2 partners	80.5% (76.2-84.7%)	75.2% (70.6-79.9%)	79.5% (74.4-84.6%)	76.7% (70.8-82.5%)	76.8% (71.0-82.6%)	0.98 (0.94-1.02)	0.386
Concurrency (≥2 current female vaginal sex partners at time of survey)	1 partner	NA	NA	NA	NA	NA	NA	NA
	≥2 partners	10.0% (6.9-13.0%)	8.4% (6.6-10.2%)	8.5% (6.0-11.0%)	8.4% (4.3-12.4%)	7.0% (4.4-9.6%)	0.97 (0.91-1.02)	0.212
								
*STI Testing*								
STI testing in past 12 months	1 partner	12.0% (10.2-13.9%)	12.7% (10.7-14.7%)	11.4% (9.7-13.2%)	12.5% (10.3-14.6%)	12.3% (10.6-14.0%)	1.00 (0.98-1.03)	0.887
	≥2 partners	32.4% (27.7-37.0%)	33.9% (29.9-37.8%)	32.7% (28.4-37.1%)	35.4% (30.0-40.9%)	27.8% (23.8-31.9%)	0.99 (0.96-1.02)	0.406

Bold indicates significance at <0.05 level.

*Estimates derived from linear regression (β). All others from logistic regression (odds ratios; OR). All models accounted for survey weights.

^Measured among Hispanic, Non-Hispanic Black, and Non-Hispanic White individuals only.

†Analysis includes 2011−13–2017−19 survey periods only. In the 2008−10 survey period, the questions about numbers of opposite-sex partners in the past 12 months for any type of sex and for specific acts were only asked if respondents reported more than 1 lifetime vaginal sex partner in the interviewer-administered portion of the survey, which led to ~20% missingness and significant bias for estimates of total, vaginal, oral, and anal sex numbers in the past 12 months.

### Sexual behaviors

#### Females.

From 2008–2010 through 2017–2019, reports of vaginal sex with a male partner in the past 12 months increased significantly among females who were married (99.1% to 99.8%) or never married (98.0% to 99.3%), and the mean number of vaginal sex acts in the past 4 weeks decreased significantly among those cohabiting (9.46 to 7.40 acts) or reporting only 1 past-year male vaginal sex partner (7.25 to 6.62 acts). From 2011–2013 through 2017–2019, oral sex with a male partner in the past 12 months increased significantly among those who were formerly married (85.2% to 92.4%), never married (85.6% to 90.7%), or reported 1 or ≥2 vaginal sex partners in the past 12 months (1 = 84.1% to 87.5%; ≥ 2 = 91.1% to 97.6%). No significant trends were observed in reported anal sex in the past 12 months.

Condom use for vaginal sex (at last sex and over past 4 weeks) decreased significantly from 2008–2010 through 2017–2019 among those who were never married or reported ≥2 partners (at last sex: never married = 51.9% to 42.9% and ≥2 partners = 45.3% to 22.6%; proportion of condom-protected sex acts in past 4 weeks: never married = 0.531 to 0.378 and ≥2 partners = 0.452 to 0.298) and also decreased significantly over the past 4 weeks among those who were cohabiting or reported only 1 partner (cohabiting = 0.212 to 0.146; 1 partner = 0.220 to 0.181). From 2011–2013 through 2017–2019, condom use at last oral sex decreased significantly only among those reporting 1 partner (6.1% to 3.9%) though similar temporal trends were observed across all strata. No trends were observed for condom use at last anal sex.

#### Males.

From 2008–2010–2017–2019, reports of vaginal sex with a female partner in the past 12 months increased (97.4% to 99.0%) and number of vaginal sex acts in the past 4 weeks decreased (4.11 to 3.40) significantly among males who had never married. From 2011–2013 to 2017–2019, oral sex in the past 12 months increased significantly among males who were cohabiting (87.5% to 93.2%) or formerly married (91.8% to 96.9%), whereas anal sex in the past 12 months decreased significantly among those who had never married (23.2% to 17.7%) or reported ≥2 past-year female vaginal sex partners (37.0% to 29.4%).

Condom use for vaginal sex decreased significantly among males who had never married or reported ≥2 partners (at last sex: never married = 61.9% to 56.3% and ≥2 partners = 54.0% to 45.7%; past 4 weeks: never married = 0.588 to 0.519 and ≥2 partners = 0.516 to 0.441). By contrast, condom use decreased significantly at last oral sex only among those currently married (3.4% to 1.2%) and did not change significantly at last anal sex among any groups.

### Sexual network attributes

#### Females.

Reporting sex in the past 12 months with a man who has had sex with other men increased significantly from 2.5% in 2008–2010 to 5.1% in 2017–2019 among females who had never married. Among cohabiting females, partnerships of the same race/ethnicity decreased significantly from 86.5% to 80.4%. No significant trends were observed in concurrency (>1 current male vaginal sex partner at time of interview) among any sub-group.

#### Males.

Reports of ever having oral or anal sex with other men increased significantly among males who were never married (3.8% to 5.5%) or who had ≥ 2 female partners (4.6% to 6.3%). Racial/ethnic homophily decreased significantly among partnerships of males who were married (88.9% to 85.9%) or never married (81.2% to 72.3%) or who had 1 partner (87.3% to 81.1%). Concurrency decreased significantly among those who were formerly married (12.4% to 7.3%).

### STI testing

Past-year chlamydia testing increased significantly from 2008 through 2010–2017–2019 among females who were married (13.0% to 17.1%), formerly married (27.6% to 45.8%), or had 1 partner (21.7% to 25.4%), but any STI testing – measured from 2011–2013–2017–2019 – only significantly increased among those who were formerly married (42.6% to 56.0%). By contrast, among males, any STI testing decreased significantly from 21.4% in 2008–2010 to 16.2% in 2017–2019 among those who were cohabiting and was stable among all other sub-groups.

Table 5 summarizes the regression estimates for all outcomes and strata. Across all analyses, models adjusting for age and race/ethnicity yielded similar results (results not shown). Supplemental Table 3 suggests that there are no temporal trends in the overall distribution of sex with opposite-sex partners (Never, Ever but none in past 12 months, 1 in past 12 months, ≥ 2 in past 12 months) [S1 File]. Supplemental Tables 4 and 5 present trends in marital/cohabiting status and partner number by age and race/ethnicity for females and males, respectively [S1 File].

**Table 5:** Estimates of the annual change in sexual behaviors, network attributes, and STI testing among females and males in the 2008−10 through 2017−19 survey periods of the National Survey of Family Growth.

	Odds ratios or β (95%CI)					
Outcome	Married	Cohabiting, not married	Divorced, widowed, or separated	Never married	1 partner	≥2 partners
*Females*						
Vaginal sex with a male partner in past 12 months	**1.20** **(1.02-1.41)**	0.92 (0.71-1.19)	1.11 (0.98-1.26)	**1.08** **(1.01-1.16)**	NA	NA
Number of vaginal sex acts with male partner(s) in past 4 weeks*	−0.016 (−0.086, 0.054)	**−0.242** **(−0.384, −0.101)**	0.037 (−0.164, 0.238)	−0.033 (−0.136, 0.070)	**−0.073** **(−0.129, −0.016)**	0.026 (−0.152, 0.204)
Proportion of condom-protected vaginal sex acts with male partner(s) in past 4 weeks*	−0.002 (−0.006, 0.001)	**−0.009** **(−0.015, −0.002)**	0.001 (−0.012, 0.014)	**−0.016** **(−0.024, −0.009)**	**−0.004** **(−0.008, −0.001)**	**−0.015** **(−0.024, −0.007)**
Condom use at last vaginal sex with a male partner	1.00 (0.97-1.03)	0.98 (0.94-1.01)	1.00 (0.95-1.05)	**0.96** **(0.93-0.98)**	0.99 (0.97-1.01)	**0.95** **(0.92-0.98)**
Had oral sex with a male partner in past 12 months	1.03 (0.99-1.07)	1.05 (0.98-1.13)	**1.14** **(1.04-1.26)**	**1.09** **(1.02-1.16)**	**1.04** **(1.00-1.07)**	**1.19** **(1.11-1.27)**
Condom use at last oral sex with a male partner^	0.90 (0.80-1.01)	0.94 (0.82-1.07)	0.92 (0.78-1.08)	0.93 (0.86-1.01)	**0.92** **(0.86-0.98)**	0.95 (0.86-1.05)
Had anal sex with a male partner in past 12 months	1.03 (0.99-1.08)	1.01 (0.95-1.08)	0.99 (0.93-1.06)	1.02 (0.97-1.07)	1.02 (0.98-1.05)	1.02 (0.97-1.08)
Condom use at last anal sex with a male partner in past 12 months	1.08 (0.96-1.22)	1.02 (0.89-1.18)	1.11 (0.96-1.29)	0.96 (0.87-1.05)	1.05 (0.98-1.13)	0.97 (0.89-1.06)
Vaginal, oral, or anal sex with a man who has sex with men in past 12 months	0.98 (0.91-1.07)	1.02 (0.92-1.15)	1.00 (0.87-1.15)	**1.09** **(1.02-1.17)**	1.02 (0.96-1.09)	1.08 (0.99-1.17)
Racial/ethnic homophily among up to 3 current male vaginal sex partners	0.99 (0.96-1.02)	**0.95** **(0.91-0.99)**	0.97 (0.92-1.03)	0.99 (0.95-1.02)	0.98 (0.95-1.00)	0.97 (0.93-1.01)
Concurrency (≥2 current male vaginal sex partners at time of survey)	1.13 (1.0-1.28)	0.94 (0.83-1.07)	1.03 (0.89-1.19)	0.99 (0.92-1.06)	NA	1.02 (0.96-1.08)
Chlamydia testing in past 12 months	**1.04** **(1.02-1.07)**	1.01 (0.97-1.04)	**1.08** **(1.04-1.13)**	1.01 (0.98-1.03)	**1.03** **(1.01-1.05)**	1.01 (0.99-1.04)
STI testing in past 12 months	1.03 (0.99-1.07)	1.02 (0.96-1.07)	**1.08** **(1.02-1.15)**	1.00 (0.96-1.05)	1.02 (1.00-1.05)	1.04 (0.99-1.10)
						
*Males*						
Vaginal sex with a female partner in past 12 months	0.94 (0.79-1.11)	1.11 (0.91-1.36)	1.09 (0.92-1.28)	**1.13** **(1.05-1.22)**	NA	NA
Number of vaginal sex acts with female partner(s) in past 4 weeks*	−0.002 (−0.066, 0.062)	−0.186 (−0.396, 0.023)	0.062 (−0.147, 0.271)	**−0.075** **(−0.142, −0.008)**	−0.032 (−0.086, 0.022)	−0.118 (−0.268, 0.032)
Proportion of condom-protected vaginal sex acts with female partner(s) in past 4 weeks*	−0.003 (−0.007, 0.001)	−0.004 (−0.012, 0.004)	0.002 (−0.014, 0.019)	**−0.010** **(−0.017, −0.004)**	−0.002 (−0.006, 0.002)	**−0.010** **(−0.016, −0.003)**
Condom use at last vaginal sex with a female partner	0.98 (0.95-1.01)	0.99 (0.95-1.03)	1.01 (0.95-1.06)	**0.97** **(0.94-0.99)**	1.00 (0.98-1.02)	**0.96** **(0.93-0.99)**
Had oral sex with a female partner in past 12 months	1.00 (0.95-1.05)	**1.10** **(1.02-1.20)**	**1.15** **(1.00-1.33)**	1.04 (0.97-1.11)	.1.03 (0.99-1.07)	1.08 (0.97-1.20)
Condom use at last oral sex with a female partner^†^	**0.86** **(0.77-0.97)**	0.99 (0.86-1.14)	1.00 (0.89-1.13)	0.96 (0.91-1.02)	0.95 (0.89-1.01)	0.93 (0.85-1.02)
Had anal sex with a female partner in past 12 months	1.03 (0.98-1.08)	1.01 (0.95-1.08)	0.98 (0.90-1.07)	**0.95** **(0.91-0.99)**	1.02 (0.98-1.06)	**0.93** **(0.89-0.98)**
Condom use at last anal sex with a female partner in past 12 months	1.00 (0.91-1.10)	1.02 (0.92-1.14)	1.07 (0.92-1.25)	0.97 (0.91-1.05)	1.03 (0.97-1.10)	0.95 (0.88-1.03)
Ever had oral or anal sex with another man	1.04 (0.99-1.08)	0.98 (0.90-1.07)	1.11 (1.00-1.23)	**1.05** **(1.00-1.11)**	1.03 (0.99-1.07)	**1.06** **(1.01-1.12)**
Racial/ethnic homophily among up to 3 current female vaginal sex partners	**0.95** **(0.91-0.99)**	0.96 (0.92-1.01)	0.93 (0.89-1.00)	**0.96** **(0.92-0.99)**	**0.94** **(0.92-0.97)**	0.98 (0.94-1.02)
Concurrency (≥2 current male vaginal sex partners at time of survey)	0.90 (0.80-1.02)	0.98 (0.87-1.11)	**0.91** **(0.84-0.99)**	0.96 (0.91-1.01)	NA	0.97 (0.91-1.02)
STI testing in past 12 months	1.02 (0.99-1.06)	**0.95** **(0.90-1.00)**	0.98 (0.93-1.04)	0.98 (0.96-1.01)	1.00 (0.98-1.03)	0.99 (0.96-1.02)

Bold indicates significance at <0.05 level. Estimates represent per-year changes from 2009 to 2018, midpoints of the 2008−10 and 2017−19 survey periods, respectively, except for oral and anal sex behaviors and any STI testing, which were measured from the 2011−13 survey period and on and represent per-year changes from 2012 to 2018.

*Estimates derived from linear regression (β). All others from logistic regression (odds ratios). All models have been weighted to represent the US household population ages 15–44.

^Among females reporting ever performing oral sex on a male partner and having had any oral sex in the past 12 months.

†Among males reporting ever receiving oral sex from a female partner and having had any oral sex in the past 12 months.

## Discussion

In a population-based sample of U.S. 15–44-year-old females and males, temporal changes in sexual behaviors with opposite-sex partners, network attributes, and STI testing from 2008–2010 through 2017–2019 varied by marital/cohabiting status and number of opposite-sex partners in the past year in complex ways. These changes have potential to decrease STI transmission (decreases in vaginal sex frequency and concurrency, increases in STI testing among females), increase transmission (increases in vaginal and oral sex, decreases in STI testing among males and condom use), or have less certain effects (decreases in racial/ethnic homophily, increases in sex with men who have sex with men/other men). Changes in sexual behaviors and networks were most common among never-married females and males and males with multiple partners, even after adjusting for age and race/ethnicity. These groups have greater likelihood of partner change [10,17] and STI acquisition [18,19], though there were fewer differences in trends when stratifying by partner number among female than males and at least one significant trend was observed in all sub-groups. In general, these trends were higher in magnitude than those observed among females and males overall in prior analyses [2,3].

Previous transmission modeling found that declines in the frequency of penile-vaginal sex and condom use and age-specific changes in STI testing among people with opposite-sex partners are likely to have accounted for only part of the increased rates of reported STI diagnoses in the 2010s[4]. Because within-group trends in our analyses were generally higher in magnitude than those stratified by sex alone and were more apparent in groups at higher risk for STI acquisition, modeling changes among females and males overall may have underestimated the potential impact of changes in these behaviors [12]. New modeling studies accounting for how relationship status and recent sexual experience affected trends in sexual behaviors, networks, and STI testing may yield more accurate estimates.

Similar analyses of 2008–2010 through 2017–2019 NSFG data stratified by age and race/ethnicity found that trends in vaginal sex behaviors, network attributes, and STI testing were most common among 20–29-year-olds, Black females and males, and White males; changes in oral and anal sex behaviors were most common among 30–44-year-old females; and White and 15–29-year-old males reported declines in opposite-sex partners [2,3]. These results intersect with ours to the extent that the likelihood of being married or previously-married increases with age and Black females and males were the most likely to report never having married and having multiple partners. However, these intersections seem not to be driving our findings given the limited temporal trends in marital/cohabiting status within age and racial/ethnic groups and no impact of adjusting for these characteristics. Yet, even stratifying by marital status and partner number may miss important sexual and relationship context for trends in sexual behaviors, networks, and STI testing. Another NSFG analysis found that condom use declined from 2002 to 2011–2017 among never- or formerly-married males ages 15–29 who had sex with females who reported STI risk factors (i.e., sex with other men; sex in exchange for money or drugs; or sex with female partner(s) who were living with HIV, injected illicit drugs, or perceived to be non-monogamous), but increased among those without such risk factors [6]. More complex analytic approaches that explore the intersectional effects of relationship, sexual, and contextual factors in relation to sexual behaviors, networks, and testing may generate additional insights into how they have contributed to increases in reported STI diagnoses.

In the U.S., chlamydia and gonorrhea screening is recommended for all sexually-active females under 25, women 25 years and older who are at increased risk, and at least annually for men who have sex with men [20,21]. Encouragingly, there were several groups of females – those who were married, previously-married, or had 1 partner – who reported increases in chlamydia testing in the past year. However, those who were married or had 1 partner may be at lower risk of STI acquisition than never-married people and those with multiple partners, among whom testing remained stable, and testing for any STI decreased among cohabiting males. In addition, even among the groups with the highest reported testing in 2017–2019 – females who had never married, previously married, or who reported multiple partners – only about half reported chlamydia testing in the past year. In prior NSFG analyses, we found that STI testing in the past year had remained stable among females and males overall from 2008–2010 through 2017–2019, but increased among females ages 30–44 and Black females and decreased among males ages 20–29 [2]. During a similar time period, other nation-wide studies found small increases in the proportion of sexually-active women ages 16–24 with at least 1 chlamydia test in the measurement year from 2011 to 2019 in both commercial and Medicaid health plans [22], but substantial decreases in chlamydia testing among young women at Title X family planning clinics [23] and declines in STD clinic visits among women and men who have sex with women [24]. The declines in testing at family planning clinics and STD clinics may have been tempered by Medicaid expansion, which was associated with relative increases in STI testing from 2011 to 2017 [25]. These data reinforce the need to substantially enhance efforts to achieve universal screening for women and to improve screening through structural interventions like expanding Medicaid, clinical public health infrastructure, and testing in other community-based settings.

Our study has several limitations. First, these self-reported outcomes may have been impacted by social desirability and recall bias, and shifting cultural attitudes over the study period, including related to same-sex behavior, may have contributed to observed changes. However, using ACASI for a subset of outcomes may have limited social desirability’s effect in these cases [26]. Second, analyses were stratified only by male vs. female. Third, marital and cohabiting status did not differentiate between same-sex and opposite-sex partners, and because both this status and past-year partner number can change over time, participant responses at the time of survey administration may not apply to the relevant recall period for all outcomes. Fourth, racial/ethnic homophily was only analyzable for Hispanic, Black, and White respondents’ three most recent, current partnerships and is therefore not representative of all partnerships. In addition, the ways partners connected changed substantially during the study period [27,28], and these data were collected prior to the COVID-19 pandemic, which impacted – at least in the short-term – sexual behaviors, networks, and STI screening [29,30]. Finally, making a large number of comparisons may have led to observing some significant differences due to chance alone, and trends may be impacted by other unmeasured changes in these groups.

Combined with previous NSFG analyses, these data present a more complete picture of trends in sexual behaviors, network attributes, and STI testing during a period of substantial increases in the incidence of reported bacterial STI diagnoses. Marital/cohabiting status and recent sexual experience provided context for previously observed trends in sexual behaviors, network attributes, and STI testing among people with opposite-sex partners. Additional research is needed to understand why behavioral and network changes are occurring in some groups but not others; how group-specific trends might be contributing to increases in STI diagnoses and persistent disparities; and which constructs, such as social determinants of health, add context and explanatory value to changes in behaviors, diagnoses, and STI disparities. All such research could be used to tailor prevention interventions and expand testing for populations disproportionately affected by STIs.

## Supporting information

S1 File Supplemental Tables 1–5. (DOCX)
