# Supplementary material for: Changes in sexual behaviors, network attributes, and STI testing among 15–44-year-olds by marital/cohabiting status and partner number: National Survey of Family Growth, 2008-19
Source: PLoS One. 2026 Apr 2;21(4):e0343813. doi: 10.1371/journal.pone.0343813 (PMC13046248; doi:10.1371/journal.pone.0343813)
Supplement: S1 File — (DOCX) [file pone.0343813.s001.docx]

**S1 File**

**Supplemental Tables 1-5 for Katz et al. Changes in sexual behaviors, network attributes, and STI testing among 15-44-year-olds by marital/cohabiting status and partner number: National Survey of Family Growth, 2008-19**

| **Supplemental Table 1. Sociodemographic characteristics of females ages 15-44 in the 2008-10 to 2017-19 survey periods of the National Survey of Family Growth** | | | | | |
| --- | --- | --- | --- | --- | --- |
|  | **2008-10** | **2011-13** | **2013-15** | **2015-17** | **2017-19** |
| **Unweighted N** | 6428 | 5601 | 5699 | 4886 | 5413 |
| **Age (y)** |  |  |  |  |  |
| 15-19 | 16.9% (15.6-18.2%) | 15.7% (14.1-17.2%) | 15.3% (14.0-16.7%) | 15.3% (13.3-17.3%) | 15.2% (13.7-16.7%) |
| 20-29 | 34.2% (31.8-36.5%) | 34.1% (32.5-35.8%) | 34.5% (32.2-36.8%) | 34.5% (32.4-36.7%) | 34.4% (32.0-36.8%) |
| 30-44 | 49.0% (46.5-51.4%) | 50.2% (47.8-52.6%) | 50.2% (47.9-52.5%) | 50.2% (48.0-52.4%) | 50.4% (48.2-52.6%) |
| **Race and ethnicity** |  |  |  |  |  |
| Hispanic/Latino | 17.3% (14.3-20.3%) | 19.7% (15.9-23.6%) | 20.3% (16.5-24.2%) | 20.8% (16.9-24.7%) | 21.3% (16.9-25.7%) |
| Non-Hispanic Black, single race | 13.7% (10.5-17.0%) | 13.9% (11.4-16.4%) | 13.5%  (10.8-16.2%) | 13.7% (10.6-16.7%) | 13.6% (10.7-16.5%) |
| Non-Hispanic White, single race | 60.8% (56.8-64.8%) | 56.9%  (52.9-61.0%) | 55.2% (51.3-59.2%) | 55.2% (50.4-60.1%) | 54.8%  (50.7-58.8%) |
| Another race/ethnicity* | 8.1% (6.6-9.7%) | 9.4% (5.5-13.2%) | 10.9% (8.8-13.0%) | 10.3% (8.8-11.9%) | 10.3% (8.6-12.1%) |
| **Education** |  |  |  |  |  |
| Less than high school diploma or GED | 24.7% (23.0-26.5%) | 19.0% (16.9-21.2%) | 18.3% (16.5-20.1%) | 18.1% (15.9-20.3%) | 17.0% (15.1-18.9%) |
| High school diploma or GED | 23.2% (21.7-24.7%) | 25.2% (23.1-27.2%) | 23.2% (20.8-25.6%) | 22.5% (20.6-24.3%) | 22.9% (20.9-24.9%) |
| Some college or associate degree | 28.8% (26.3-31.3%) | 30.3% (27.9-32.7%) | 29.8% (27.6-31.9%) | 30.6% (28.0-33.1%) | 30.7% (29.3-32.2%) |
| Bachelor's degree or higher | 23.2% (20.8-25.7%) | 25.5% (22.3-28.6%) | 28.7% (25.5-31.9%) | 28.9% (25.4-32.3%) | 29.3% (26.3-32.3%) |
| **Federal poverty level** |  |  |  |  |  |
| <138% | 33.9% (31.5-36.4%) | 36.1% (33.4-38.8%) | 35.7% (32.9-38.6%) | 33.9% (31.7-36.1%) | 33.1% (30.2-36.0%) |
| ≥138% | 66.1% (63.6-68.5%) | 63.9% (61.2-66.6%) | 64.3% (61.4-67.1%) | 66.1% (63.9-68.3%) | 66.9% (64.0-69.8%) |
| **Health insurance status** |  |  |  |  |  |
| Private only | 60.7% (57.7-63.6%) | 57.1% (53.6-60.5%) | 61.6% (58.2-65.0%) | 61.2% (58.1-64.3%) | 62.0% (58.7-65.2%) |
| Any public | 19.0% (16.8-21.2%) | 22.3% (19.4-25.2%) | 23.3% (21.0-25.6%) | 25.3% (22.3-28.3%) | 25.4% (22.9-27.9%) |
| None, IHS only, or single service only | 20.3% (18.5-22.2%) | 20.6% (18.3-22.9%) | 15.1% (12.8-17.4%) | 13.5% (11.4-15.6%) | 12.7% (10.2-15.1%) |
| **Marital and cohabiting status** | | | | | |
| Married | 39.4% (37.0-41.7%) | 38.1% (35.0-40.4%) | 38.1% (36.0-40.2%) | 37.8% (35.4-40.1%) | 35.4% (33.3-37.5%) |
| Cohabiting, not married | 12.2% (10.8-13.5%) | 15.0% (13.5-16.5%) | 14.6% (13.1-16.1%) | 13.8% (11.9-15.7%) | 14.7% (13.2-16.3%) |
| Divorced, widowed, or separated | 9.7% (8.6-10.8%) | 9.1% (8.0-10.2%) | 7.6% (6.7-8.5%) | 7.7% (6.7-8.8%) | 6.5% (5.6-7.4%) |
| Never married | 38.8% (36.4-41.2%) | 37.8% (35.2-40.4%) | 39.7% (37.5-41.9%) | 40.7% (37.9-43.6%) | 43.4% (41.1-45.7%) |
| GED = General Education Diploma. IHS = Indian Health Service.  All estimates have been weighted to represent the US household population ages 15-44.  *Includes non-Hispanic individuals reporting multiple races. | | | | | |

| **Supplemental Table 2. Sociodemographic characteristics of males ages 15-44 in the 2008-10 to 2017-19 survey periods of the National Survey of Family Growth** | | | | | |
| --- | --- | --- | --- | --- | --- |
|  | **2008-10** | **2011-13** | **2013-15** | **2015-17** | **2017-19** |
| **Unweighted N** | 5538 | 4815 | 4506 | 3998 | 4622 |
| **Age (y)** |  |  |  |  |  |
| 15-19 | 17.2% (16.0-18.5%) | 16.7% (15.3-18.1%) | 16.2% (14.9-17.6%) | 16.1% (14.6-17.5%) | 15.9% (14.2-17.6%) |
| 20-29 | 34.2% (32.1-36.4%) | 34.4% (31.8-37.0%) | 34.9% (32.6-37.1%) | 34.9% (31.7-38.1%) | 34.8% (31.0-38.5%) |
| 30-44 | 48.5% (46.5-50.5%) | 48.9% (46.7-51.2%) | 48.9% (46.3-51.5%) | 49.0% (46.1-52.0%) | 49.3% (46.0-52.7%) |
| **Race and ethnicity** |  |  |  |  |  |
| Hispanic/Latino | 19.5% (16.2-22.8%) | 21.1% (17.4-24.7%) | 21.4% (17.2-25.7%) | 21.9% (17.9-25.8%) | 22.3% (18.1-26.5%) |
| Non-Hispanic Black, single race | 11.9% (9.1-14.8%) | 11.7% (9.4-14.1%) | 12.1% (9.6-14.5%) | 12.3% (9.7-14.8%) | 12.3% (10.0-14.7%) |
| Non-Hispanic White, single race | 60.1% (56.4-63.8%) | 56.7% (52.8-60.6%) | 56.6% (52.3-61.0%) | 56.0% (51.5-60.5%) | 54.7% (50.3-59.1%) |
| Another race or ethnicity* | 8.5% (6.9-10.0%) | 10.5% (7.8-13.2%) | 9.9% (7.6-12.1%) | 9.9% (8.3-11.4%) | 10.7% (8.5-13.0%) |
| **Education** |  |  |  |  |  |
| Less than high school diploma or GED | 30.9% (28.6-33.2%) | 22.2% (20.1-24.2%) | 22.7% (20.5-24.9%) | 19.4% (17.7-21.2%) | 17.4% (15.1-19.6%) |
| High school diploma or GED | 23.2% (21.4-24.9%) | 28.8% (26.5-31.1%) | 26.8% (24.5-29.2%) | 25.4% (23.0-27.8%) | 29.2% (26.3-32.1%) |
| Some college or associate degree | 26.3% (24.3-28.3%) | 27.5% (24.8-30.1%) | 28.1% (26.2-30.0%) | 28.0% (25.6-30.3%) | 27.6% (24.5-30.7%) |
| Bachelor's degree or higher | 19.6% (17.6-21.7%) | 21.6% (18.2-25.0%) | 22.4% (19.6-25.2%) | 27.2% (23.8-30.6%) | 25.8% (23.3-28.4%) |
| **Federal poverty level** |  |  |  |  |  |
| <138% | 26.9% (24.7-29.1%) | 28.1% (25.4-30.9%) | 26.4% (24.3-28.4%) | 24.4% (21.9-26.9%) | 22.6% (20.1-25.0%) |
| ≥138% | 73.1% (70.9-75.3%) | 71.9% (69.1-74.6%) | 73.6%  (71.6-75.7%) | 75.6% (73.1-78.1%) | 77.4% (75.0-79.9%) |
| **Health insurance status** |  |  |  |  |  |
| Private only | 60.4% (57.5-63.4%) | 57.2% (53.8-60.6%) | 62.9% (60.1-65.6%) | 64.9% (61.8-68.1%) | 65.5% (62.0-69.1%) |
| Any public | 13.0% (11.7-14.3%) | 17.6% (14.7-20.6%) | 16.9% (14.8-19.0%) | 17.1% (14.7-19.6%) | 19.2% (16.8-21.5%) |
| None, IHS only, or single service only | 26.5% (24.0-29.0%) | 25.2% (22.5-27.8%) | 20.2% (18.2-22.2%) | 17.9% (15.5-20.3%) | 15.3% (13.1-17.6%) |
| **Marital and cohabiting status** | | | | | |
| Married | 36.3% (34.2-38.4%) | 35.2% (31.8-38.6%) | 37.0% (34.6-39.4%) | 35.9% (32.5-39.2%) | 34.9% (32.0-37.8%) |
| Cohabiting, not married | 12.6% (11.2-14.0%) | 13.5% (11.7-15.3%) | 13.3% (11.8-14.8%) | 12.3% (10.5-14.1%) | 12.6% (11.0-14.2%) |
| Divorced, widowed, or separated | 5.4% (4.3-6.5%) | 5.7% (4.9-6.4%) | 4.6% (3.9-5.2%) | 3.7% (3.2-4.3%) | 4.0% (3.2-4.8%) |
| Never married | 45.7% (43.7-47.8%) | 45.6% (43.1-48.1%) | 45.2% (42.5-47.8%) | 48.1% (45.2-51.0%) | 48.6% (45.2-51.9%) |
| GED = General Education Diploma. IHS = Indian Health Service.  All estimates have been weighted to represent the US household population ages 15-44.  *Includes non-Hispanic individuals reporting multiple races. | | | | | |

| **Supplemental Table 3. Sex with opposite-sex sex partners* among all females and males in the 2008-10 to 2017-19 survey periods of the National Survey of Family Growth** | | | | | | | | | | | |
| --- | --- | --- | --- | --- | --- | --- | --- | --- | --- | --- | --- |
|  | | **2008-10** | | **2011-13** | | **2013-15** | | **2015-17** | | **2017-19** | |
| **Females** | | | | | | | | | | | |
| Never had sex with male partner | 10.5% (9.3-11.6%) | | 9.5% (8.0-11.1%) | | 10.3% (9.2-11.5%) | | 10.5% (8.9-12.2%) | | 11.5% (10.2-12.8%) | |  |
| Ever had sex with male partner, but none in past 12 months | 7.3% (6.3-8.3%) | | 8.2%  (7.1-9.3%) | | 7.9% (6.8-9.0%) | | 6.6% (5.6-7.5%) | | 8.5% (7.2-9.9%) | |  |
| 1 male partner in past 12 months | 68.3% (66.6-70.0%) | | 67.7% (65.3-70.0%) | | 68.0% (66.2-69.9%) | | 67.1% (64.9-69.3%) | | 66.0% (64.2-67.8%) | |  |
| ≥2 male partners in past 12 months | 14.0% (12.2-15.7%) | | 14.6% (12.9-16.3%) | | 13.7% (12.5-14.9%) | | 15.8% (14.4-17.3%) | | 14.0% (12.6-15.4%) | |  |
| **Males** | | | | | | | | | | | |
| Never had sex with female partner | | 11.5% (10.6-12.5%) | | 11.2% (9.8-12.6%) | | 10.5% (9.1-11.8%) | | 13.1% (11.6-14.6%) | | 13.4% (11.9-14.9%) | |
| Ever had sex with female partner, but none in past 12 months | | 7.7% (6.6-8.8%) | | 6.9% (6.0-7.9%) | | 6.5% (5.6-7.4%) | | 7.3% (6.1-8.5%) | | 7.6% (6.3-8.9%) | |
| 1 female partner in past 12 months | | 61.8% (60.0-63.8%) | | 63.0% (60.7-64.5%) | | 63.8% (61.7-65.9%) | | 62.1% (59.9-64.2%) | | 63.0% (60.6-65.4%) | |
| ≥2 female partners in past 12 months | | 19.0%  (17.3-20.8%) | | 18.8% (17.2-20.5%) | | 19.2% (17.3-21.0%) | | 17.6% (15.9-19.3%) | | 16.0% (14.7-17.3%) | |
| All estimates have been weighted to represent the US household population ages 15-44. *Includes anal, oral, or vaginal sex. | | | | | | | | | | | |

| **Supplemental Table 4. Marital/cohabiting status and number of male vaginal sex partners in past 12 months among females ages 15-44, stratified by age and race and ethnicity, in the 2008-10 to 2017-19 survey periods of the National Survey of Family Growth** | | | | | | | | |
| --- | --- | --- | --- | --- | --- | --- | --- | --- |
|  |  | **% or Mean (95% Confidence Interval)** | | | | | **Multinomial or logistic regression*** | |
|  |  | **2008-10** | **2011-13** | **2013-15** | **2015-17** | **2017-19** | **OR (95%CI)** | **p-value** |
| *Age* | *Marital/cohabiting Status* |  |  |  |  |  |  |  |
| 15-19 years | Married | 1.2%  (0.5-2.0%) | 1.3%  (0.7-2.6%) | 0.7%  (0.0-1.4%) | 0.7%  (0.1-1.2%) | 0.2%  (0.0-0.4%) | NA | |
|  | Cohabiting, not married | 4.5%  (3.1-5.9%) | 5.1%  (2.8-7.3%) | 3.8%  (2.2-5.3%) | 3.0%  (0.8-5.3%) | 2.2%  (0.5-3.8%) | 0.92  (0.70-1.21) | 0.562 |
|  | Divorced, widowed, or separated | - | - | - | - | 0.0%  (0.0-0.1%) | NA | |
|  | Never married | 94.3%  (92.6-96.0%) | 93.7%  (91.1-96.2%) | 95.5%  (93.9-97.1%) | 96.3%  (93.9-98.7%) | 97.6%  (96.0-99.2%) | Reference | |
| 20-29 years | Married | 27.9%  (24.9-31.0%) | 27.8%  (24.4-31.0%) | 25.2%  (21.9-28.5%) | 25.7%  (22.0-29.3%) | 21.7%  (19.0-24.5%) | **0.96**  **(0.93-0.98)** | **0.002** |
|  | Cohabiting, not married | 19.2%  (16.6-21.9%) | 24.2%  (21.3-27.1%) | 22.5%  (19.6-25.5%) | 21.5%  (17.5-25.5%) | 19.8%  (17.1-22.6%) | 0.98  (0.96-1.01) | 0.172 |
|  | Divorced, widowed, or separated | 5.0%  (3.1-6.8%) | 3.4%  (2.4-4.4%) | 3.8%  (2.7-4.9%) | 2.7%  (1.9-3.4%) | 2.7%  (1.9-3.5%) | **0.92**  **(0.87-0.97)** | **0.002** |
|  | Never married | 47.9%  (43.4-52.4%) | 44.7%  (40.9-48.4%) | 48.4%  (44.3-52.6%) | 50.1%  (45.8-54.4%) | 55.7%  (52.4-59.0%) | Reference | |
| 30-44 years | Married | 60.5%  (57.4-63.6%) | 56.7%  (52.5-60.9%) | 58.4%  (55.4-61.4%) | 57.4% (54.3-60.5%) | 55.4%  (51.9-58.8%) | **0.96**  **(0.93-0.98)** | **<0.001** |
|  | Cohabiting, not married | 9.9%  (8.3-11.4%) | 11.8%  (9.7-13.9%) | 12.4%  (10.4-14.9%) | 11.7% (9.8-13.6%) | 15.0%  (12.3-17.7%) | 1.00  (0.97-1.04) | 0.834 |
|  | Divorced, widowed, or separated | 16.4%  (14.4-18.3%) | 15.9%  (13.8-17.9%) | 12.5%  (10.9-14.1%) | 13.6%  (11.5-15.7%) | 11.0%  (9.2-12.7%) | **0.93**  **(0.90-0.95)** | **<0.001** |
|  | Never married | 13.2%  (11.4-15.1%) | 15.7%  (13.0-18.3%) | 16.7%  (14.4-18.9%) | 17.3%  (14.8-19.8%) | 18.6%  (16.2-21.0%) | Reference | |
|  |  |  |  |  |  |  |  |  |
| *Race and ethnicity* | *Marital/cohabiting Status* |  |  |  |  |  |  |  |
| Hispanic/Latino | Married | 37.8%  (33.9-41.7%) | 32.6%  (28.4-36.7% | 36.6%  (33.8-39.3%) | 35.6% (30.3-41.0%) | 32.1%  (28.0-36.3%) | **0.96**  **(0.94-0.99)** | **0.009** |
|  | Cohabiting, not married | 17.3%  (14.7-19.9%) | 19.6%  (17.2-22.0%) | 15.9%  (13.1-18.7%) | 13.6% (10.1-17.1%) | 16.8% (12.4-21.2%) | **0.96**  **(0.92-0.99)** | **0.023** |
|  | Divorced, widowed, or separated | 9.0%  (7.1-11.0%) | 11.8%  (8.8-14.8%) | 8.1%  (5.8-10.3%) | 8.3%  (5.7-10.8%) | 7.6%  (5.8-9.3%) | **0.95**  **(0.91-0.98)** | **0.004** |
|  | Never married | 35.9%  (33.1-38.6%) | 36.0% (31.7-40.4%) | 39.5%  (36.7-42.3%) | 42.5%  (37.1-47.8%) | 43.5% (39.5-47.5%) | Reference | |
| Non-Hispanic Black, single race | Married | 21.7%  (17.2-26.1%) | 20.3%  (16.9-23.8%) | 19.4% (15.5-23.3%) | 19.0% (13.7-24.3%) | 18.1%  (14.2-22.0%) | 0.97  (0.94-1.01) | 0.164 |
|  | Cohabiting, not married | 10.1%  (7.7-12.6%) | 9.4%  (7.2-11.6%) | 12.7%  (9.8-15.5%) | 11.9% (8.7-15.0%) | 13.1%  (9.8-16.4%) | 1.03  (0.98-1.07) | 0.240 |
|  | Divorced, widowed, or separated | 11.7%  (9.4-13.9%) | 12.9%  (9.7-16.1%) | 7.8%  (5.8-9.7%) | 9.1%  (6.0-12.2%) | 8.4%  (6.1-10.8%) | **0.95**  **(0.91-0.99)** | **0.009** |
|  | Never married | 56.6%  (52.5-60.6%) | 57.3%  (53.9-60.6%) | 60.1% (55.7-64.6%) | 60.1% (55.0-65.1%) | 60.4%  (55.2-65.6%) | Reference | |
| Non-Hispanic White, single race | Married | 44.5%  (41.2-47.8%) | 45.0%  (41.4-48.6%) | 42.6% (40.0-45.2%) | 43.7% (40.8-46.5%) | 39.9%  (12.5-17.2%) | **0.98**  **(0.95-1.00)** | **0.021** |
|  | Cohabiting, not married | 11.3% (9.9-12.7%) | 14.7% (12.4-17.0%) | 15.3%  (13.2-17.3%) | 14.1%  (11.4-16.9%) | 14.8%  (12.5-17.2%) | 1.01  (0.98-1.04) | 0.504 |
|  | Divorced, widowed, or separated | 9.8% (8.3-11.4%) | 7.7%  (12.4-17.0%) | 7.5%  (6.1-8.9%) | 6.9%  (5.5-8.2%) | 5.6%  (4.5-6.7%) | **0.93**  **(0.90-0.96)** | **<0.001** |
|  | Never married | 34.4%  (31.0-37.8%) | 32.7%  (29.8-35.5%) | 34.6%  (32.2-37.1%) | 35.3% (32.2-38.4%) | 39.6% (36.8-42.4%) | Reference | |
| Another race or ethnicity^†^ | Married | 34.6%  (29.3-39.8%) | 34.4% (20.6-48.1%) | 41.6% (34.3-48.9%) | 35.3%  (27.5-43.0%) | 41.1% (34.0-48.2%) | 1.04  (0.98-1.09) | 0.182 |
|  | Cohabiting, not married | 11.2%  (7.3-15.0%) | 15.2% (8.9-21.6%) | 11.2%  (7.3-15.0%) | 14.6%  (9.0-20.3%) | 12.1%  (7.6-16.5%) | 1.02  (0.96-1.09) | 0.473 |
|  | Divorced, widowed, or separated | 6.8%  (4.3-9.3%) | 6.6% (3.7-9.5%) | 6.7% (3.8-9.7%) | 9.5%  (5.2-13.8%) | 6.2%  (3.1-9.2%) | 1.03  (0.96-1.10) | 0.418 |
|  | Never married | 47.5% (41.4-53.5%) | 43.8%  (33.2-54.4%) | 40.5% (33.9-47.0%) | 40.6% (32.9-48.3%) | 40.6% (32.9-48.4%) | Reference | |
|  |  |  |  |  |  |  |  |  |
| *Age* | *Partner Number* |  |  |  |  |  |  |  |
| 15-19 years | 1 partner | 56.9%  (50.2-63.6%) | 62.1% (55.8-68.4%) | 61.7%  (54.3-69.1%) | 64.0% (55.8-72.1%) | 59.1% (51.8-66.4%) | Reference | |
|  | ≥2 partners | 43.1%  (36.4-49.8%) | 37.9% (31.6-44.2%) | 68.3% (30.9-45.7%) | 36.1%  (27.9-44.2%) | 40.9% (33.6-48.2%) | 0.98  (0.94-1.03) | 0.452 |
| 20-29 years | 1 partner | 78.2%  (75.4-81.0%) | 78.5% (75.5-81.4%) | 78.3% (75.2-81.3%) | 73.1% (69.3-76.9%) | 76.6%  (73.0-80.3%) | Reference | |
|  | ≥2 partners | 21.8% (19.0-24.6%) | 21.5% (18.6-24.5%) | 21.7% (18.7-24.8%) | 26.9% (23.1-30.7%) | 23.4% (19.7-27.0%) | 1.02  (1.00-1.05) | 0.114 |
| 30-44 years | 1 partner | 92.1% (90.3-93.8%) | 89.5% (87.3-91.8%) | 90.4% (89.0-91.7%) | 90.3% (88.6-91.9%) | 91.0% (89.5-92.4%) | Reference | |
|  | ≥2 partners | 7.9% (6.2-9.7%) | 10.5% (8.2-12.7%) | 9.6%  (8.3-11.0%) | 9.7% (8.1-11.4%) | 9.0%  (7.6-10.5%) | 1.01  (0.98-1.04) | 0.471 |
|  |  |  |  |  |  |  |  |  |
| *Race and ethnicity* | *Partner Number* |  |  |  |  |  |  |  |
| Hispanic/Latino | 1 partner | 78.1% (74.5-81.7%) | 82.5%  (79.3-85.8%) | 77.7% (72.6-82.8%) | 78.7% (74.2-83.1%) | 78.8% (78.9-82.7%) | Reference | |
|  | ≥2 partners | 21.9%  (18.3-25.5%) | 17.5%  (14.2-20.7%) | 22.3% (17.2-27.4%) | 21.3% (16.9-25.8%) | 21.2%  (17.3-25.1%) | 1.02  (0.99-1.06) | 0.253 |
| Non-Hispanic Black, single race | 1 partner | 61.6% (57.3-66.0%) | 67.8% (63.6-71.9%) | 61.6%  (54.4-68.7%) | 61.4% (55.0-67.9%) | 66.1% (58.4-73.8%) | Reference | |
|  | ≥2 partners | 38.4% (34.0-42.7%) | 32.2% (28.1-36.4%) | 38.4% (31.3-45.6%) | 38.6% (32.1-45.0%) | 33.9% (26.2-41.6%) | 0.99  (0.97-1.02) | 0.615 |
| Non-Hispanic White, single race | 1 partner | 79.3% (76.8-81.9%) | 79.7% (76.6-82.8%) | 80.3% (77.8-82.7%) | 83.3% (80.5-86.2%) | 83.8% (82.0-85.6%) | Reference | |
|  | ≥2 partners | 20.7% (18.1-23.2%) | 20.3% (17.2-23.4%) | 19.7% (17.3-22.2%) | 16.7% (13.8-19.5%) | 16.2%  (14.4-18.0%) | 1.01  (0.98-1.04) | 0.487 |
| Another race or ethnicity^†^ | 1 partner | 86.9% (82.2-91.6%) | 80.1% (72.9-87.2%) | 85.2% (80.0-90.4%) | 82.4% (75.7-89.2%) | 86.9% (81.8-92.0%) | Reference | |
|  | ≥2 partners | 13.1% (8.4-17.8%) | 19.9% (12.8-27.1%) | 14.8% (9.6-20.0%) | 17.6% (10.8-24.3%) | 13.1% (8.0-18.2%) | 1.00  (0.94-1.06) | 0.860 |
| All estimates have been weighted to represent the US household population ages 15-44. *Estimates for marital/cohabiting status from multinomial regression and for partner number from logistic regression. ^†^Includes non-Hispanic individuals reporting multiple races. | | | | | | | | |

| **Supplemental Table 5. Marital/cohabiting status and number of female vaginal sex partners in past 12 months among males ages 15-44, stratified by age and race and ethnicity, in the 2008-10 to 2017-19 survey periods of the National Survey of Family Growth** | | | | | | | | |
| --- | --- | --- | --- | --- | --- | --- | --- | --- |
|  |  | **% or Mean (95% Confidence Interval)** | | | | | **Multinomial or logistic regression*** | |
|  |  | **2008-10** | **2011-13** | **2013-15** | **2015-17** | **2017-19** | **OR (95%CI)** | **p-value** |
| *Age* | *Marital/cohabiting Status* |  |  |  |  |  |  |  |
| 15-19 years | Married | 0.5% (0.0-1.3%) | 0.6% (0.1-1.0%) | 0.1% (0.0-0.2%) | 0.1%  (0.0-0.2%) | 0.1% (0.0-0.2%) | 0.77  (0.0.64-0.95) | 0.014 |
|  | Cohabiting, not married | 2.8% (1.4-4.1%) | 1.8% (0.8-2.7%) | 0.7% (0.2-1.2%) | 1.4% (0.0-2.8%) | 0.9% (0.3-1.6%) | 0.88  (0.79-0.98) | 0.018 |
|  | Divorced, widowed, or separated | - | - | - | 0.3%  (0.0-1.0%) | - | NA | |
|  | Never married | 96.7% (95.2-98.2%) | 97.6% (96.6-98.7%) | 99.2% (98.7-99.7%) | 98.2% (96.6-99.7%) | 99.0% (98.3-99.7%) | Reference | |
| 20-29 years | Married | 21.5% (18.4-24.7%) | 19.8% (15.0-24.6%) | 20.5% (17.2-23.7%) | 20.4% (16.1-24.7%) | 18.6%  (15.2-21.9%) | 0.98  (0.95-1.01) | 0.201 |
|  | Cohabiting, not married | 17.5%  (14.1-20.9%) | 18.7% (15.5-21.9%) | 18.9% (16.4-21.5%) | 14.9%  (11.6-18.2%) | 16.8% (13.3-20.3%) | 0.98  (0.94-1.02) | 0.243 |
|  | Divorced, widowed, or separated | 2.0% (1.3-2.7%) | 2.0% (1.2-2.8%) | 2.2% (1.4-3.0%) | 1.4% (0.7-2.2%) | 1.3% (0.7-2.0%) | 0.95  (0.90-1.00) | 0.066 |
|  | Never married | 59.0%  (55.1-62.9%) | 59.5% (54.8-64.2%) | 58.4% (54.6-62.2%) | 63.3% (58.9-67.6%) | 63.3%  (58.0-68.6%) | Reference | |
| 30-44 years | Married | 59.3% (56.0-62.7%) | 57.8% (53.4-62.2%) | 61.0% (57.8-64.2%) | 58.6% (54.7-62.5%) | 57.5% (53.9-61.2%) | 0.98  (0.95-1.00) | 0.61 |
|  | Cohabiting, not married | 12.7%  (10.4-14.9%) | 13.9% (11.3-16.4%) | 13.4% (10.9-15.9%) | 14.1% (11.6-16.5%) | 13.4% (11.1-15.6%) | 0.98  (0.95-1.02) | 0.311 |
|  | Divorced, widowed, or separated | 9.7% (7.5-11.9%) | 10.2% (8.7-11.7%) | 7.7%  (6.5-9.0%) | 6.4% (5.2-7.7%) | 7.1% (5.6-8.7%) | **0.93**  **(0.90-0.97)** | **<0.001** |
|  | Never married | 18.3% (15.7-21.0%) | 18.1% (15.5-20.7%) | 17.8% (15.5-20.2%) | 20.9% (18.4-23.4%) | 22.0% (19.5-24.4%) | Reference | |
|  |  |  |  |  |  |  |  |  |
| *Race and ethnicity* | *Marital/cohabiting Status* |  |  |  |  |  |  |  |
| Hispanic/Latino | Married | 32.7%  (28.8-36.5%) | 34.9%  (29.5-40.2%) | 33.9% (29.5-38.3%) | 34.9% (28.8-41.0%) | 33.4% (29.9-37.0%) | 0.99  (0.96-1.02) | 0.383 |
|  | Cohabiting, not married | 19.3% (16.1-22.5%) | 18.4%  (15.3-21.4%) | 17.4% (13.0-21.8%) | 15.4% (10.8-20.0%) | 14.0% (10.1-17.9%) | **0.95**  **(0.91-0.99)** | **0.013** |
|  | Divorced, widowed, or separated | 5.3% (3.1-7.5%) | 3.9% (2.5-5.3%) | 3.8% (2.4-5.1%) | 3.5% (2.4-4.6%) | 3.9% (2.4-5.4%) | 0.95  (0.89-1.02) | 0.125 |
|  | Never married | 42.7%  (38.4-47.1%) | 42.9%  (37.8-47.9%) | 45.0% (39.8-50.1%) | 46.2% (40.2-52.2%) | 48.6% (44.6-52.6%) | Reference | |
| Non-Hispanic Black, single race | Married | 25.2% (21.7-28.7%) | 26.3% (21.2-31.4%) | 27.3% (21.7-32.9%) | 23.8% (17.5-30.1%) | 23.5% (18.8-28.1%) | 1.00  (0.97-1.02) | 0.901 |
|  | Cohabiting, not married | 12.4% (9.3-15.4%) | 15.4% (11.3-19.6%) | 13.7% (9.8-17.6%) | 11.4% (7.9-14.9%) | 11.9% (8.2-15.6%) | 1.02  (0.99-1.04) | 0.307 |
|  | Divorced, widowed, or separated | 5.3% (3.4-7.2%) | 4.7% (3.3-6.1%) | 3.8% (2.5-5.1%) | 4.8% (2.6-6.9%) | 6.2% (3.8-8.6%) | **0.95**  **(0.91-0.98)** | **0.006** |
|  | Never married | 57.1% (53.0-61.2%) | 53.6% (48.7-58.6%) | 55.2% (49.1-61.3%) | 60.0% (52.0-68.0%) | 58.5% (53.0-64.0%) | Reference | |
| Non-Hispanic White, single race | Married | 38.8% (35.8-41.7%) | 38.1% (33.8-42.5%) | 40.3% (37.2-43.4%) | 39.0% (34.7-43.3%) | 38.4% (33.9-43.0%) | 0.98  (0.95-1.02) | 0.379 |
|  | Cohabiting, not married | 10.6% (8.9-12.3%) | 11.3% (9.1-13.5%) | 12.6% (10.7-14.5%) | 12.0% (9.6-14.3%) | 12.3% (10.3-14.4%) | 0.98  (0.94-1.08) | 0.356 |
|  | Divorced, widowed, or separated | 5.8% (4.1-7.4%) | 6.6% (5.4-7.8%) | 5.2% (4.3-6.2%) | 3.6% (2.9-4.3%) | 4.0% (3.0-5.0%) | 1.01  (0.94-1.08) | 0.814 |
|  | Never married | 44.8% (41.9-47.8%) | 43.9% (41.0-46.9%) | 41.9% (38.7-45.1%) | 45.5% (41.8-49.1%) | 45.3% (40.6-50.0%) | Reference | |
| Another race or ethnicity^†^ | Married | 42.3% (35.9-48.7%) | 29.8% (20.7-38.9%) | 36.8% (29.3-44.4%) | 35.3% (26.6-43.9%) | 32.9% (25.2-40.5%) | 0.96  (0.92-1.01) | 0.128 |
|  | Cohabiting, not married | 11.8% (6.7-16.9%) | 13.5% (6.7-20.4%) | 8.0% (4.7-11.3%) | 8.6% (5.8-11.5%) | 11.6% (7.3-16.0%) | 0.96  (0.89-1.03) | 0.261 |
|  | Divorced, widowed, or separated | 3.1% (0.5-5.6%) | 5.4% (2.2-8.7%) | 3.4% (1.5-5.3%) | 3.7% (1.7-5.6%) | 1.6% (0.6-2.5%) | 0.92  (0.85-1.00) | 0.053 |
|  | Never married | 42.8% (36.0-49.7%) | 51.3% (44.4-58.1%) | 51.8% (45.0-58.7%) | 52.4% (45.2-59.7%) | 53.9% (45.1-62.8%) | Reference | |
|  |  |  |  |  |  |  |  |  |
| *Age* | *Partner Number* |  |  |  |  |  |  |  |
| 15-19 years | 1 partner | 50.0% (43.8-46.3%) | 58.3% (51.8-64.8%) | 56.3% (50.5-62.1%) | 59.2% (53.2-65.1%) | 62.0% (55.6-68.5%) | Reference | |
|  | ≥2 partners | 50.0% (43.7-56.3%) | 41.7% (35.2-48.2%) | 43.7% (37.9-49.5%) | 40.8% (34.9-46.8%) | 38.0%  (31.5-44.4%) | **0.95**  **(0.92-0.99)** | **0.013** |
| 20-29 years | 1 partner | 69.9% (66.4-73.3%) | 70.5% (66.3-74.8%) | 66.8% (62.3-71.2%) | 69.5% (64.7-74.2%) | 73.7% (70.4-77.0%) | Reference | |
|  | ≥2 partners | 30.1% (26.7-33.6%) | 29.5% (25.2-33.7%) | 33.2% (28.8-37.7%) | 30.5% (25.8-35.3%) | 26.3% (23.0-29.6%) | 0.99  (0.96-1.01) | 0.308 |
| 30-44 years | 1 partner | 86.6% (84.3-89.0%) | 87.5% (85.0-90.0%) | 88.1% (86.2-89.9%) | 88.2% (86.1-90.4%) | 87.8% (85.6-90.0%) | Reference | |
|  | ≥2 partners | 13.4% (11.0-15.7%) | 12.5% (10.0-15.0%) | 11.9% (10.1-13.8%) | 11.8% (9.6-13.9%) | 12.2% (10.0-14.4%) | 0.99  (0.96-1.02) | 0.370 |
|  |  |  |  |  |  |  |  |  |
| *Race and ethnicity* | *Partner Number* |  |  |  |  |  |  |  |
| Hispanic/Latino | 1 partner | 78.1% (74.5-81.7%) | 82.5% (79.3-85.8%) | 77.7% (72.6-82.8%) | 78.7% (74.2-83.1%) | 78.8%  (74.9-82.7%) | Reference | |
|  | ≥2 partners | 21.9% (18.3-25.5%) | 17.5% (14.2-20.7%) | 22.3% (17.2-27.4%) | 21.3% (16.9-25.8%) | 21.2% (17.3-25.1%) | 1.01  (0.97-1.04) | 0.742 |
| Non-Hispanic Black, single race | 1 partner | 61.6% (57.3-66.0%) | 67.8% (63.6-71.9%) | 61.6% (54.4-68.7%) | 61.4% (55.0-67.9%) | 66.1% (58.4-73.8%) | Reference | |
|  | ≥2 partners | 38.4% (34.0-42.7%) | 32.2% (28.1-36.4%) | 38.4% (31.3-45.6%) | 38.6% (32.1-45.0%) | 33.9% (26.2-41.6%) | 0.99  (0.96-1.03) | 0.735 |
| Non-Hispanic White, single race | 1 partner | 79.3% (76.8-81.9%) | 79.7% (76.6-82.8%) | 80.3% (77.8-82.7%) | 83.3% (80.5-86.2%) | 83.8% (82.0-85.6%) | Reference | |
|  | ≥2 partners | 20.7% (18.1-23.2%) | 20.3% (17.2-23.4%) | 19.7% (17.3-22.2%) | 16.7% (13.8-19.5%) | 16.2% (14.4-18.0%) | **0.97**  **(0.94-0.99)** | **0.002** |
| Another race or ethnicity^†^ | 1 partner | 86.9% (82.2-91.6%) | 80.1% (72.9-87.2%) | 85.2% (80.0-90.4%) | 82.4% (75.7-89.2%) | 86.9% (81.8-92.0%) | Reference | |
|  | ≥2 partners | 13.1% (8.4-17.8%) | 19.9% (12.8-27.1%) | 14.8% (9.6-20.0%) | 17.6% (10.8-24.3%) | 13.1% (8.0-18.2%) | 0.99  (0.94-1.05) | 0.805 |
| All estimates have been weighted to represent the US household population ages 15-44. *Estimates for marital/cohabiting status from multinomial logistic regression and for partner number from logistic regression. ^†^Includes non-Hispanic individuals reporting multiple races. | | | | | | | | |
